# Mental illness, forced labour, and colonial biopower in Kabba Province of Northern Nigeria, 1900–1947

**DOI:** 10.1017/mdh.2025.10045

**Published:** 2026-04

**Authors:** Unekwu Friday Itodo

**Affiliations:** History and International Studies, Prince Abubakar Audu University, Nigeria

**Keywords:** Colonialism, Biopower, Exploitation, Forced Labour, Human Rights, Kabba Province, Marginalisation, Mental Illness, Northern Nigeria

## Abstract

Examining the systemic exploitation of mentally ill individuals, this study focuses on the practices of the British colonial administration in Kabba Province, Northern Nigeria (1900–1947). This research investigates how colonial authorities employed biopolitical strategies to categorise, control, and exploit this vulnerable population for labour, prioritising colonial economic and administrative interests. The study utilises a qualitative methodology, primarily analysing archival documents from the National Archives of Nigeria (NAK), Kaduna, and Arewa House Archives (AHA), to uncover the forced labour system’s practices and rationalisations. Crucially, it incorporates oral sources from direct descendants of ethno-medical practitioners, former colonial staff, traditional chiefs, and learned community members. This oral history component provides vital intergenerational knowledge, contextualising archival findings and offering perspectives often absent from official records, ensuring a nuanced understanding of pre-colonial mental health practices and colonial-era lived experiences. Secondary literature on colonial biopower, mental health history, and regional history provides a comparative framework. Findings indicate the colonial administration systematically repurposed traditional care and established new mechanisms to identify, isolate, and compel mentally ill individuals into various forms of forced labour for infrastructure and economic extraction. In conclusion, this research significantly contributes to scholarship on vulnerable populations during colonialism, illuminating the intricate link between mental illness, labour, and power in colonial Nigeria, and informing contemporary debates on mental health, human rights, and historical justice.

## Introduction

The area that is now Kogi State in Northern Nigeria was formerly Kabba Province, established by the British in 1900.^1^ The province was divided into four regions with Lokoja as the capital.^2^ The dominant ethnic groups shared similar customs and resided in a tropical climate with distinct wet and dry seasons.^3^ The population of the province grew significantly over time, from 181,557 in 1925,^4^ 460,895 in 1940,^5^ and increased slightly to 551,398 after the Second World War in 1948.^6^ Mirroring the exploitation across Africa, the colonial regime in Kabba Province imposed a brutal system of forced labour. Forced labour, a cornerstone of human exploitation throughout history, robs individuals of their free will and compels them to work under threat of violence or punishment.^7^ This abhorrent practice manifested in various forms, from chattel slavery to debt bondage, often preying on vulnerable populations with limited options. During this era of colonialism, forced labour became a central tool for economic exploitation. European powers, driven by insatiable greed for resources and profit from their colonies, implemented a range of coercive schemes to mobilise a subjugated workforce.^8^ These schemes often involved brutal systems of manipulation of existing hierarchies to coerce local populations into providing labour for mines, plantations, or infrastructure projects.^9^

The devastating consequences of colonial forced labour extended far beyond the physical health of those subjected to it. The gruelling nature of the work, coupled with inadequate living conditions and often barbaric punishments, created a constant state of fear and anxiety. This relentless pressure chipped away at the psychological well-being of the labourers.^10^ In this environment, mental illnesses, already a complex and multifaceted issue, were undoubtedly exacerbated by the inhumanity and relentless pressure of colonial forced labour. The debate on colonial mental health practices has ranged from the impact of colonial mental health practices on African individuals and communities, the relevance of Western psychiatric models in African contexts, and the role of cultural understandings in shaping mental health practices. Leland argues that colonial mental health practices caused psychological trauma, marginalisation, and limited access to appropriate care for Africans.^11^ Similarly, Sandosky contends that the colonial approach to mental health in Nigeria, characterised by a lack of understanding of local context and the imposition of Western medical practices, had a profound and lasting negative impact on the mental well-being of Nigerians.^12^ Kilroy-Marac delves into the intersection of post-colonial psychiatry and memory work in a West African clinic in Senegal, emphasising the clinic’s role in reconstructing the past, making sense of it, and using it to shape the present and future.^13^ Quarshie explores the link between mental distress and enslavement in Atlantic-era West Africa, highlighting the role of Ga shrines as healing spaces for mentally distressed individuals. She sheds light on the practice of spiritual pawning by priests, who converted mentally ill individuals into potential subjects of enslavement.^14^ Ochiai examines the emergence of asylums for mental patients in Africa during the mid-nineteenth century. He argues that while colonial authorities were initially reluctant to establish these institutions due to financial constraints and limited facilities, they eventually felt compelled to seclude individuals with problematic behaviour to maintain social order and protect against perceived threats from the insane.^15^ Heaton’s work further illuminates the complexities of colonial psychiatry in Nigeria, particularly in his analysis of migration, mental illness, and the repatriation of ‘lunatics’, highlighting the extensive measures taken by the colonial administration to control and categorise those deemed mentally unsound.^16^

While existing scholarship, notably from authors such as Quarshie, Sadowsky, and the collective works in Hunt and Büschel, illuminated the complex history of mental illness in Africa, the specific intersection of mental health conditions and forced labour during the colonial era, particularly in Northern Nigeria, has received limited attention. This neglect has significant implications for our understanding of the complexities and challenges of mental health on the continent and within the broader colonial project. This study aimed to address this lacuna by exploring the interconnectedness between mental health and forced labour in Northern Nigeria. By focusing on this specific region, which has been understudied in mental health scholarship, it contributes to a broader understanding of the complex interplay between these two issues in Africa. To address this scholarly lacuna, this study argues that the British colonial administration in Kabba Province, Northern Nigeria, employed a system of biopower that exploited vulnerable individuals suffering from mental illness. This argument rests on two key pillars: forced labour and economic exploitation. Colonial authorities, motivated by economic gain, saw mentally ill individuals as a readily available source of cheap labour. This exploitation manifested in work on government projects in the prisons and sanitation.

This study contributes to a wider scholarship on mental health and forced labour by drawing parallels with similar historical experiences in other parts of the world during the same period. Scholars like Peter Barham have explored the racialised and imperial dimensions of understanding and treating mental illness, a context that resonates with the colonial experience in Nigeria.^17^ The exploitation of patient labour, a critical aspect of this study, finds echoes in Monk’s work on Kew Cottages, Australia,^18^ and Pinto’s examination of lunatic asylums in the Bombay Presidency, demonstrating a global pattern of instrumentalising vulnerable populations for economic gain within institutional settings.^19^ Furthermore, Wallis’s work on how medical professionals viewed patient bodies, considering their ‘muscle wastage’ and ‘degenerate mass’ in 19th-century asylums, underscores the medical gaze applied to those deemed insane, often linking physical capacity to moral worth, a perspective that undoubtedly informed the colonial administration’s assessment of the labour potential of the mentally ill.^20^ Stephen Snelders’ research on forced labour and leprosy in Suriname highlights how marginalised groups, including those with stigmatised health conditions, were systematically coerced into labour by colonial regimes.^21^ Crucially, this study’s historical analysis of colonial exploitation aligns with and contributes to contemporary efforts, such as those within ‘Mad Studies’ articulated by Peter Beresford and Diana Rose, to decolonise global mental health by uncovering and challenging the enduring legacies of oppressive psychiatric systems.^22^ By examining these connections, this essay not only enriches the history of mental health in Northern Nigeria but also positions it within a comparative framework, illustrating how the colonial state’s use of forced labour of individuals with mental illness was part of a broader, global imperial strategy to control bodies and extract resources.

## Colonialism, prisons, and mental health patients

In Northern Nigeria, long before colonial influence and even in the early colonial era, the burden of caring for individuals with mental illness rested upon their families and communities.^23^ Mental health challenges were understood through three primary lenses: natural, preternatural, and mystical forces. This holistic view dictated how these conditions were approached and treated by families and the community, who bore the primary responsibility for the affected individual.^24^ Families often managed the health care of their loved ones by either using their own knowledge of herbal remedies or by seeking out specialised experts.^25^ This dual approach is exemplified by the Igala people, where while professional herbalists, known as *obochi*, existed, every individual, especially the respected elders *abogujo*, possessed some medicinal knowledge about plants and trees.^26^ When a family’s own knowledge was insufficient, they would look for specialists outside their community, such as Wada Enoh of Umomi, who was widely known for treating mental illness. In this system, families displayed a remarkable level of self-reliance in caring for their loved ones. This extended beyond basic medical knowledge to include a unique ability to calm patients. Even when a patient was stubborn or violent, their family members or caregivers could intervene and, through their words alone, bring about a sense of peace. In cases where a patient had no direct family, the traditional community of Kabba Province, where everyone was considered family, would assign an individual or a rotating group of people from the community to care for the patient until they regained consciousness or, in the worst-case scenario, died.^27^ Isolation, unlike the later colonial asylum model, was generally not the primary approach.^28^

While the British government established a protectorate in 1900, their control faced challenges like the Kano Rebellion of 1903.^29^ However, a decisive victory against remaining resistance in Borno in 1906 solidified British control over the entire region.^30^ The effective occupation of the people led to their exploitation for the benefit of the imperial state through forced labour in prisons. These prisons held individuals on spurious charges including assault, trespass, wounding, disobedience to order, escape, public intoxication, malicious injury to property, attempt to commit felony, and attempted suicide.^31^ The prisons were located in the provincial capitals where Europeans primarily resided. In instances where the provincial headquarters’ location changed, the prisons also moved with the Europeans to the new location. There, the prison served the dual purpose: keeping out of circulation those deemed harmful to Europeans and utilising prisoners for the benefit of Europeans by having them produce food and maintain their environment.^32^

Nevertheless, it is germane to note that forced labour was not exclusive to colonial practices. European populations also faced it, exemplified by over 70,000 French convicts sent to Guyana between 1852 and 1945, most of whom never returned.^33^ However, its application in British (and broader European) colonial contexts developed distinct specificities regarding scale, purpose, and underlying legal and racial frameworks, aimed to mobilise vast indigenous populations for large-scale infrastructure, resource extraction, and agriculture.^34^ Notorious examples include the British Colonial administration enforcing forced labour through arbitrary and discriminatory legal frameworks, bypassing established norms common to European convicts. These included ‘Lazy Native’ Laws, which targeted indigenous people deemed ‘idle’ or ‘vagrant’.^35^ Taxation, such as hut or poll taxes payable only in cash, directly compelled Africans into wage labour: for instance, the hut tax in Natal Colony contributed 75% of all Natal revenues by 1849.^36^

This was also very prominent in the province.^37^ In 1932, Miles Clifford, the District Officer of Igala Division, opposed a reduction in taxes in the hope that this would curb laziness. He stated, ‘the increased demand would tend to combat the natural laziness of the Ankpa Igala and force them to cultivate all area larger than that which barely sufficed for their physical needs’.^38^ The tax was so that women with visible taxable income were also taxed and in 1956 the women protested the tax.^39^ A critical colonial specificity backing the stated was the racialised justification for forced labour, often rationalised through pseudo-scientific theories of racial hierarchy and the ‘civilizing mission’. Indigenous populations were depicted as ‘lazy’ or ‘backward’, purportedly needing forced labour for ‘development’ into the modern capitalist economy.^40^ This narrative framed coercion as paternalistic upliftment, obscuring economic motives and human rights abuses, distinguishing it from punishment for individual transgressions applied to European convicts.^41^ Colonial prisons and detention centres often directly funnelled individuals into forced labour gangs, blurring lines between punishment and economic exploitation,^42^ a practice that was not limited to the sane but also included the mentally ill, who were particularly vulnerable to abuse and neglect. The most egregious form of this exploitation was economic, often manifesting as forced labour. The mentally ill, stripped of their dignity and humanity, were subjected to forced labour, toiling in the fields under the relentless sun.

Although the establishment of the Lokoja Lunacy Asylum in the 1920s^43^ was initially intended as a beacon of hope for the mentally ill, it was tragically transformed into a house of horrors under colonial rule. While the asylum’s founding may have seemed benevolent, the underlying economic motivations of colonialism set the stage for exploitation. However, it is important to note that this practice of exploiting the mentally ill for labour did not begin with the asylum, even though it became more prevalent there. At the inception of colonialism, Lokoja, the first capital of Northern Nigeria and the capital of Kabba Province, had an established prison that also housed individuals with mental illness from across the province.^44^ This prison served as a cornerstone of British colonial control in Northern Nigeria. Beyond its role in punishing criminals, the prison was strategically employed to quell resistance against colonial rule. Individuals who dared to challenge British authority, from local leaders to ordinary citizens, found themselves incarcerated. Furthermore, and most importantly, the prison provided a captive workforce for the demanding labour needs of colonial projects. Prisoners were subjected to gruelling tasks, constructing infrastructure and supporting the economic interests of the colonial administration.^45^ This dual function of punishment and forced labour solidified British dominance while exploiting the local population.

Just a year after solidifying control over Northern Nigeria, the British enacted the 1907 Lunacy Ordinance for Southern Nigeria.^46^ This ordinance laid the groundwork for the establishment of the Yaba Lunacy Asylum in 1907, which initially housed only 48 patients.^47^ In contrast to this development in the South, a direct parallel was not established in the North.^48^ This is because, prior to 1914, Northern and Southern Nigeria were separate entities in the traditional and political sense. This divide persisted until 1914 when the British merged them into a single colony, laying the groundwork for a unified Nigeria despite their distinct traditional experiences.^49^

Thus, in the North, the 1907 law was not effective. The Northern Nigeria colonial government, which was medically dismissive^50^ and downplayed diseases like yellow fever, even when they obviously existed,^51^ also disregarded mental health issues and used patients for labour. The British takeover of Northern Nigeria came with the established network of prisons that served as agricultural hubs, particularly focused on cultivating cash crops like cotton and groundnuts. Britain’s motivation for this system was twofold: securing a steady source of raw materials for their industries and generating revenue from the colony. To incentivise cash crop production among the local population, the government and European companies offered support, such as providing seeds and other agricultural resources. Prison farmlands, typically established on government-owned territories, produced both food for sustenance and cash crops for export. While not all prisons participated in this cash crop initiative at the beginning of colonialism, several locations like Zungeru in Niger Province, Nassarawa Province, and Lokoja in Kabba Province became hubs for cultivating crops like cotton, cocoa, and even soybeans, depending on the region’s suitability, through prison labour.^52^ A stark example of this system in action before amalgamation, aside from Lokoja, was Ankpa Prison within Kabba Province. Here, individuals deemed ‘lunatics’ were forced to contribute to agricultural production, cultivating yams and Indian corn (maize) alongside other prisoners.^53^

In 1916, after the amalgamation of Northern and Southern Nigeria, the Lunacy Ordinance was enacted to cover entire Nigeria, introducing a standardised approach to managing mental illness across Nigeria.^54^ This legislation established asylums for the mentally ill and outlined procedures for involuntary commitment and discharge. The native court, however, was given power to order mentally ill persons to Asylum, and in such a case, the procedure is for the magistrate to adjudicate Section 13 of the lunacy ordinance and issue an order if necessary.^55^ Discharge from the Asylum was by the magistrate’s order under Section 17 of the lunacy ordinance. Virtually, therefore, such persons were confined on the certificate of one medical practitioner and discharged on the recommendation of two.^56^ Essentially, the 1916 Lunacy Ordinance marked a significant shift from the ad hoc methods of managing mental illness in pre-colonial Northern Nigeria to a formalised system with defined legal procedures and institutions. Despite the 1916 Lunacy Ordinance mandating confinement in asylums for the mentally ill, Northern Nigeria lacked such an institution until the 1920s. This legislative gap left a tragic reality unchanged. Individuals suffering from mental illness remained confined within the harsh environment of native authority and provincial prisons, rather than receiving specialised care in an Asylum.

It should be noted that the native authority prisons haboured most of the mentally ill persons.^57^ Lokoja Provincial Prison, which housed a contingent of ‘non-criminal’ mentally ill individuals, was designated as the primary facility for the province’s ‘Criminal Lunatics’. This arrangement was necessitated by the inadequate facilities of native authority prisons to accommodate such inmates. A notable case of a ‘Criminal Lunatic’ in the prison was that of Onuche of Aihi, committed to Lokoja Convict Prison in August 1918 for homicide.^58^ Aside from the provincial prison, the native authority prisons lacked facilities and, as such, housed only ‘non-dangerous lunatics’ which purpose is for labour.^59^ At the Agbaja prison farm, all efforts were directed towards cotton production, and mentally ill individuals were made to labour in the fields.^60^ At the close of 1922, Captain F. Byng-Hall reported that the Lokoja convict prison held a staggering 128 inmates, subjected to forced labour.^61^ Within these prison facilities, convicts also performed various maintenance tasks to keep their surroundings clean. This included sweeping markets and roads, contributing to the overall hygiene of the prison town. Additionally, at the beginning of the year, the Koton Karfe prison initiated a program where inmates were employed in soap making.^62^

In 1926, Captain F. Byng-Hall reported that Lokoja convict prison housed a surprising mix of inmates. While some were prisoners convicted of crimes, a significant portion, 32 in all, were classified as ‘lunatics’.^63^ Among the 32 lunatics, there were ‘criminal lunatics’, those who committed crimes due to mental illness. This hints at a system that acknowledged mental illness but did not have a separate treatment structure. Interestingly, the report also mentions 10 non-criminal lunatics. Their presence alongside prisoners was motivated by a need for labour. Prisoners and lunatics were employed in cleaning tasks and prison farms, blurring the lines between punishment and treatment for mental illness.^64^ During that same year, Northern Nigeria government prisons held a total of 170 mentally ill persons, encompassing both those deemed criminals and non-criminals. Here is the breakdown of the figures (Table 1).

**Table 1.** Lunatics in native authority’s prison in Northern Nigeria, 1926

Province	Number
Adamawa	6
Bauchi	3
Bornu	10
Benue	10
Kabba Province (Lokoja)	32
Kano	11
Niger	2
Plateau	19
Sokoto	25
Zaria	52
**Total**	170

*Source:* NAK.SNP 17/12224/extract from the Annual Report on the Prisons Department, Northern Nigeria, for the year 1928.

A troubling detail emerges from these data on mentally ill persons detained in Northern Nigerian prisons. While all provinces held some mentally ill persons, both criminal and non-criminal, Kabba Province housed a significantly higher number (32) than others, which is the second largest after Zaria. This suggests systemic exploitation in the region.

Lokoja, strategically located at the confluence of the Niger and Benue Rivers, was a colonial trade hub.^65^ Its importance attracted traders, officials, and a substantial workforce.^66^ This influx of people created a demand for labour that was often met through exploitation. Individuals, including those with mental health issues, were coerced or imprisoned to fulfil these demands.^67^ The prison in Lokoja served as a convenient source of cheap, captive labour, contributing to the disproportionately high number of inmates.^68^ Even so, the prisoners were not properly treated or fed, highlighting the systemic mistreatment of vulnerable populations during the colonial era. Inspection notes of the Lokoja prison by Wynne Davis, the senior medical officer of the province in 1925, reveal the condition of all the inmates in the native authority prison in Lokoja. The note states, ‘the prisoners complained of a monotony and insufficiency in diet. They stated and this was borne out by the Dogarai that their sole diet is dry roasted yam. They got no soup, or change to maize, ground nuts, cassava, or other food stuffs in their season, neither is there any meat even supplied. Seeing that they are doing and are expected to do hard manual work, I think the diet…. are restricted to, is quite insufficient. They have two meals a day’.^69^ This was despite being assigned various tasks, beyond the prison grounds, including carrying mail and transporting loads for government officials.^70^

## Founding the Northern Nigeria Central ‘Lunatic Asylum’ Lokoja

The lunacy asylum is a historical institution specifically designed to house and care for those suffering from mental illness, particularly those considered severely afflicted.^71^ The planned asylum for the northern provinces, deviating from its purported purpose of humane care, starkly illuminates the fundamental truth that the colonial administration’s motive in health care was unequivocally *not* for the interest of the colonised people. Rather, medical provisions were instrumentalised, serving as a coercive tool to maintain colonial control, exploit resources, and safeguard European personnel. This reality is encapsulated by the brutal description of medical services in the mid-1930s as a ‘barrack service’, a system designed to ‘fend for the Europeans and the natives in government employment’, a clear indication of its utilitarian and discriminatory nature.^72^ This ‘barrack service’ was inherently strategic. The health of European administrators, soldiers, and traders was paramount for the sustained functioning of the colonial apparatus. Similarly, the health of the indigenous labour force, be it for mines, plantations, or infrastructure projects, was critical for economic output and imperial stability. Their well-being was viewed through a lens of productivity, not human rights.

Historian Helen Tilley has meticulously documented this instrumental approach. Tilley’s concept of Africa as a ‘living laboratory’ vividly portrays how scientific and medical research was undertaken not out of benevolent curiosity but to address imperial concerns, such as understanding diseases that threatened European settlers or the productivity of the African workforce.^73^ Disease control, therefore, became a mechanism for social engineering and economic efficiency, securing colonial power rather than serving the health needs of the broader population.^74^ Megan Vaughan further asserts that colonial medicine was deeply enmeshed in the construction of the ‘African subject.’ Medical discourse often framed African illnesses, including mental health conditions, in ways that justified colonial intervention and control, frequently linking pathology to perceived ‘backwardness’ or ‘social disintegration’.^75^ The establishment of institutions like asylums, therefore, was less about genuine therapeutic care and more about managing and containing individuals deemed disruptive or unprofitable to the colonial order. These facilities served to remove ‘undesirable’ elements, reinforcing governmental interests and control rather than promoting holistic well-being.^76^

The founding of the asylum aligned with the colonial approach; it was more about removing them from society ‘taking them out of circulation’ and putting them to labour, than providing proper treatment. While similar efforts in Abeokuta, Southern Nigeria, envisioned a more traditional asylum model, the planned asylum in the north appears to be a repurposed prison structure for managing ‘Dangerous Lunatics’, contrasting with the potentially more therapeutic approach planned for Abeokuta.^77^

The plan to use the Lokoja prison structures exposed a fundamental flaw in the proposed asylum system. Prisons are designed for punishment and confinement, not treatment or rehabilitation. This approach becomes even more concerning when considering the suggestion for the continuous use of the prison and its environment, and its effect on the people living in the surrounding areas. The search for an asylum for the mentally ill in Northern Nigeria unfolded through a series of proposals and revisions. Initially, the prison director favoured Zungeru as the location. However, the lieutenant governor and medical authorities disagreed, due to a lack of facilities and engagement for the mentally ill persons. The officials then proposed converting the main part of Lokoja convict prison into an asylum because the mentally ill persons could be gainfully engaged in cotton work.^78^ A site visit by the lieutenant governor revealed a potential problem: the existing prison annex was too close to the proposed asylum. This led to a new recommendation – dedicating the entire Lokoja prison to house mentally ill patients.^79^ Following the visit to the Lokoja prison, the government initially designated cells 1 to 8 and 14 as the Lunacy Asylum. However, after structural alterations within the prison it was no longer suitable. Therefore, a revised location for the asylum was declared. This new designated area encompasses Ward 1 (Cells 1 to 13) and Ward 2 within the main prison enclosure, along with Ward 3 located in the annex.^80^ After the takeover of the prison, Sir Herbert Richmond Palmer, the Lieutenant Governor, on 15 September 1926, directed H. O. Wright, the Acting Secretary for the Northern Province, to enquire about the number of mentally ill persons housed in Lokoja. A response revealed a population of 30 mentally ill persons: 27 males and 3 females. The breakdown categorised nine mentally ill persons as criminals and the remaining 21 as non-criminals, while 25 were tagged not dangerous and 5 were tagged dangerous, with two of the latter being female.^81^

However, even as the government officials strove to establish the Lunacy Asylum at Lokoja, discontent simmered among the European population. This sentiment culminated in a conference held on 1 July 1927, where influential figures like the assistant surveyor general, senior sanitary officer for Northern provinces, and senior medical officer voiced their strong objections. The primary concern centred around the undesirable proximity of the asylum to European residences, hinting at anxieties about safety, hygiene, or the social stigma associated with mental illness. The Europeans vehemently rejected the idea of a combined ‘prison cum asylum’ facility, advocating for a clear physical separation between the prison and the asylum. Overall, the Europeans’ objections prioritised their own comfort and safety over addressing the needs of the mentally ill.^82^

G. Mille Clifford, the Station Magistrate in Lokoja, emerged as a strong voice against the proposed Lunacy Asylum. In a letter dated 28 January 1927, he expressed his disapproval, stating his disappointment that the project was still being considered. The Magistrate’s primary concerns differed from those of the European residents. He focused on the logistical difficulties, arguing that Lokoja’s location was far from ideal for most provinces. He envisioned a large influx of patients requiring transport, a process he deemed arduous and inefficient. The Magistrate outlined the challenges of transferring mentally ill persons – train journeys, inadequate holding facilities in Minna, and cramped launch rides to Lokoja. While the Station Magistrate’s motivations remain unclear, his objections highlighted the practical challenges of managing a large patient population at a central Asylum with poor accessibility.^83^ According to G. Mille Clifford,

‘It is no exaggeration to state that so many halts and changes tend to unsettle the mental condition of even a normal person; and that the difficulty of keeping lunatics under restraints at the various stopping places and on the journeys must be considerable. The probable disturbance of those who live or work at the several places, and of those who may have to travel as passengers with lunatics also deserve consideration’.^84^

G. Mille Clifford stated further that the purely local objection to the scheme may be stated as:

The proposed asylum is very close to the bungalow of the superintendent of prisons, and is not far from the residence of other European officials. Although there are only about 32 lunatics now in the prison, yet the noise of their howls at night is very unpleasant and disturbing, even to the occupant of my bungalow which is not the nearest. If the number of lunatics is increased, it is reasonable to expect that the noise will be still greater; and I submit it is not right that on the hot, breathless nights which last for months in Lokoja the rest of the station should be disturbed by the screeches of a large lunatic asylum.^85^

Adding to the chorus of disapproval, J. C. Sciortino the resident of the province (the most senior government official) expressed concerns that the action was a show of incivility. He states:

I am in full agreement with the station magistrate, and in fact I would go further. I submit that if such a proposal were made in a civilised country, viz., to set up a lunatic asylum close to the residence of ordinary citizens, there would probably be legal action taken to prevent such a step. In this country it is impossible to take such legal action, and therefore on behalf of all the officials who have to live and work close to the Lokoja prison, I appeal against the proposal to convert it into a lunatic asylum. The demented howls of two or three of the more violent lunatics now confined there are distressing enough. Increase in the number of lunatic and life in Lokoja-or at any rate in the European residential quarters will become a nightmare.^86^

Despite facing this multifaceted opposition, the governor ultimately disregarded these concerns and proceeded with the Lokoja Asylum. The acting chief secretary to the Northern government wrote, ‘I am directed by the governor to inform that his excellency has considered the situation as regards the accommodation of a number of Lunatics in Lokoja jail which his Excellency’s opinion is unsatisfactory and should, in any case, be regarded and dealt with as a purely local problem.’^87^ The Lokoja prison, which was made up of mostly cells, with only a kitchen, guard room, warder’s office, and pavilion, was declared Asylum.^88^ The establishment unveiled a troubling narrative. While the government’s actions might have been driven by pragmatism and resource constraints, the chosen path prioritised cost-effectiveness and expediency at the severe expense of basic human rights and the well-being of both the mentally ill population and the staff entrusted with their care**.**

## The asylum system in Northern Nigeria: a paradox

**Figure 1.**
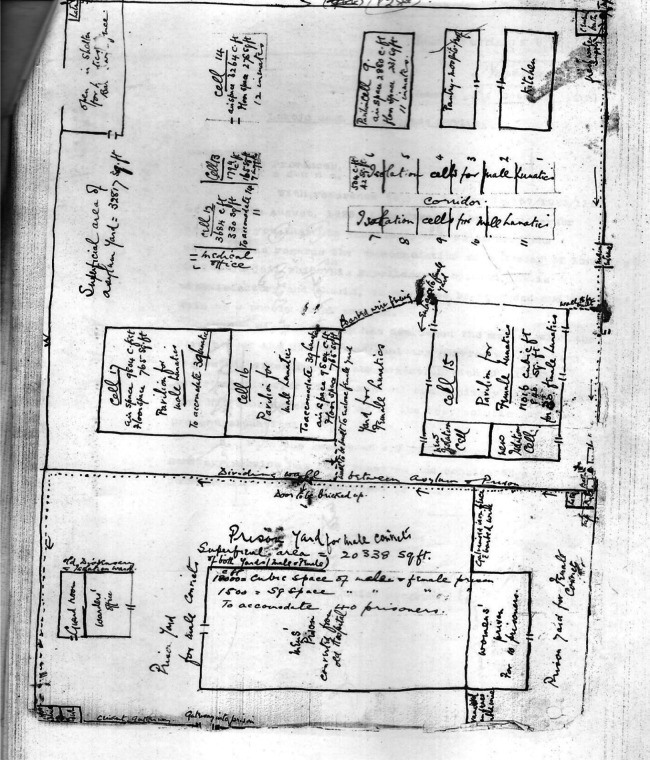
Map of the Lokoja prison turned into Asylum. *Source:* NAK/SNP. 17. 12235. Vol. 1/Lokoja Lunatic Asylum, 1928, p. 7.

The establishment of the so-called ‘Lunacy Asylum’ in Lokoja, Northern Nigeria, during the colonial era presents a complex and troubling story. (Figure 1).

The sketched map of the North Central Lunacy Asylum reveals the colonial administration’s clear prioritisation of containment over the welfare of its mentally ill inmates. Beyond a few administrative and service areas, a kitchen, guard room, warder’s office, and a pavilion, the asylum’s layout consisted almost entirely of cells. This emphasis on confinement over care is further evidenced by the decision to repurpose the existing prison hospital not for the asylum, but as a new provincial prison (see footnote 22),^89^ to accommodate both mentally ill persons and prisoners.

Prisons in the region were harsh institutions designed to punish offenders.^90^ The living conditions were difficult.^91^ Hard labour was a common punishment, and some prisoners were even shackled with leg irons.^92^ Native courts handled the cases of the prisoners sent to prison and all districts had native courts. For instance, the Igala division in the province with 29 districts had 29 native courts.^93^ Meaning, most criminal cases went through trials in native courts.^94^ The 1936 annual reports put it aptly: ‘A careful scrutiny is made of every case which comes before a native court and a large number of cases are carefully reviewed and sometimes revised by district officer. Everybody sentenced to imprisonment by a native court has the opportunity of appealing to an administrative officer before undergoing his sentence.’^95^ The possibility of appeals suggests a form of recourse.

However, the system’s harshest reality was the treatment of people deemed ‘lunatics’.^96^ In the Lokoja asylum, these individuals were kept alongside convicts and categorised as ‘Dangerous’ or ‘Non-Dangerous’ and ‘Criminal’ or ‘Non-Criminal’.^97^ Without a trial, they faced a complete lack of proper care and understanding. Where prisoners were convicted, dangerous and criminal ‘lunatics’ were offenders in the form of nuisance, noise, and, in a few cases, murders, non-criminal and non-dangerous ‘lunatics’ were only certified and imprisoned. In this case, they were ‘harmless, wandering and idiot children with underdeveloped heads’.^98^ ‘They include an idiot and other person with unsound mind.’^99^ Thus, they were easily used for labour due to the scarcity of labour and their perceived docility. According to historian Abiye Ichaba, ‘the colonial drive for infrastructure, production of cash crops, that led to hunger for labour, turned to a surprising source: mentally ill patients. Non-violent mental patients, seen as docile due to their condition, were deemed suitable for manual labour. This policy, fuelled by the need for manpower, filled colonial coffers at a human cost.’^100^ Ichaba’s assertion regarding the exploitation of mentally ill patients for forced labour in colonial contexts is well-supported by scholarly research on colonial labour practices and the history of psychiatric institutions in the imperial period. For instance, Vaughan provides extensive evidence of the instrumentalisation of medical institutions, including asylums, to serve colonial economic and administrative aims.^101^ This instrumentalisation took various forms across the empire; notably, in contexts such as South Africa, political opponents were sometimes pathologised, termed ‘lunatics’, and subsequently imprisoned,^102^ while in regions like Kabba Province of Northern Nigeria, a particularly submissive area to colonial authority, the primary victims of this forced labour within asylums were often individuals with pre-existing mental vulnerabilities.

The targeted group was not the ‘dangerous lunatics’ who might pose a threat, but rather harmless individuals with intellectual disabilities. These people were easy to control and lacking the mental capacity to resist. While ‘dangerous lunatics’ were unfit for manual labour due to unpredictable behaviour, these targeted individuals were used because they were perfect for exploitation. This exemplifies the dehumanising practices of colonialism, where human beings were seen as expendable resources to fuel imperial greed. Mentally ill persons in the asylum were exploited through forced labour, a system associated with slavery and repression, and was a tool of British imperialism in Africa.^103^ According to Okia,

‘Essentially, if Africans would not work for Europeans, which was in their best interests anyway; they must be forced to work. Out of this moral and economic “dilemma” of development grew the multi-headed Hydra of forced labour in Africa: forced labour for private interests, government forced labour and coercion in the interests of community.’^104^

Forced labour was the major source of labour during the colonial era and the government exploited all available means to make it available. Walter Rodney echoed the same view. In his words,

‘The simplest form of forced labour was that which colonial governments exacted to carry out “public works”. Labour for a given number of days per year had to be given free for these “public works” – building castles for governors, prisons for Africans, barracks for troops, and bungalows for colonial officials. A great deal of this forced labour went into the construction of roads, railways and ports to provide the infrastructure for private capitalist investment and to facilitate the export of cash-crops.’^105^

The government at first used conscription for labour, but it proved inadequate. The government then resorted to coercion. Consequently, natives were forcibly recruited by the sophisticated colonial state and private interests into working in businesses and government areas, thereby reducing the cost of labour by placing the onus of the colonial labour force on coercion.^106^

Prisons were major contributors to colonial labour. Colonial prisons in Northern Nigeria took this venture to another level by adding mentally ill persons to the colonial labour force, and worst was the Lokoja Asylum. A 1937 memo revealed the deplorable conditions in asylums. Written by a medical officer to the registrar of the Kaduna High Court, this document unveiled the shocking reality behind the walls where mentally ill individuals were confined. The memo relates to the case of Rex vs. Martin, where the court deemed the defendant unfit for trial due to unsound mind. The court ordered his confinement in the Lokoja prison, a decision John. P. Harrison, the medical officer, strongly contested. He argued that, ‘With reference to the extract from the medical evidence, I should like to invite your attention to the following statement- “with good nursing and expert treatment he might in time get better”, and to point out that such nursing is not possible in Lokoja asylum where lunatics are confined in the government prison. However excellent the buildings may be as a prison, they are totally unsuitable for the good nursing that is recommended for a possible recoverable case.’^107^

Before that time, even the governor was not satisfied with what he saw in the asylum on his visits to the province. The following extract from W. O. P Rosedale, the resident of Kabba Province’s notes on his Excellency’s visit in September 1936 indicates his view: ‘his excellency appeared to disapprove of lunatics being confined in a prison, and while he seemed satisfied with the establishment as a prison, he was far from satisfied that (a) the building was suitable for the accommodation of lunatics or (b) that criminals and lunatics should be confined in the same building’.^108^ Briercliffe, the director of medical services, reported after the inspection of the asylum by Dr. McGrath and Mr. A. Clark, assistant prison superintendent, on 12 and 13 December 1938, that ‘the kitchen building, the dark single cells, the high prison walls… would have to be demolished’.^109^ He further states that the mentally ill persons ‘at present are living under the worst possible conditions, and they are without better accommodation and trained staff’.^110^ Even worse, the warders lack the necessary training to take care of the mentally ill, yet the population of the mentally ill increased from 1927. By 1936, the mentally ill persons in the Lokoja Central Lunacy Asylum were 39, of which 34 were male and 5 were female.^111^

These mentally ill persons were used to clear European stations during the rainy season.^112^ In Lokoja, where the asylum was located, mosquitoes and sandflies were numerous at all times of the year owing to the extensive breeding grounds among the swamps on the foreshore below high-water levels and siltation in the dry season, which dams the outlet of watercourses. The township, both the commercial part of it and the slum area, needed to be continuously and strictly taken care of.^113^ The immediate strategy for malaria control was based on the elimination of all actual and potential breeding places for mosquitoes. This was the prime concern of the local public health authorities in Lokoja, where vast seasonal *anopheline* breeding could and should be prevented. Mentally ill persons were used to achieve malaria control through the permanent and cheapest method of malaria control: filling, levelling, and draining most, if not all, pits and depressions in which standing water collects. The mentally ill also fill most of the borrow pits by using the spoil from the old city wall.^114^

Mentally ill persons were also used to produce clothes and other cotton-related work in the asylum.^115^ As an inspection note by J. Hampton, the senior health officer (S.H.O) between 3 and 7 January 1941, put it, ‘with the exception of the few who must be kept under permanent restraint, all the lunatics are usefully employed in cotton ginning and spinning’.^116^ The note continued in another paragraph, ‘excellent heavy clothes is being produced for uniform and “cloth”. Bonemeal in quantity. There is a very good garden, and “composting” (three months from first to last pit) is carried on with absence of flies and smells’.^117^ On 24 August 1941, J. Hampton reported again, ‘the lunatics happy and occupied except for some 8-9 dangerous lunatics who have to be confined but even they are now in the open air, under cover, all day instead of being chained in cell. There are signs not only of organisation here and in the goal but of an individual having sympathy and understanding. Prisoners are being taught mason’s work, carpentry, bookmaking, tailoring and the female lunatics spin cotton which is woven by men. All clothing is made in the two institutions’.^118^

This shows clearly why the colonial government deliberately kept the ‘not dangerous’ or ‘non-criminal’ mentally ill in the asylum. Supplementary Table 2 shows that the number of mentally ill non-criminals in the Lunacy Asylum as of 1936 was 39.^119^ The Lokoja Lunacy Asylum alone provided the colonial government with 39 labourers without reward. The above was the reason why no single mentally ill person was discharged from the asylum. As a 1936 memo puts it: ‘the discharge rates among mentally ill persons are practically nil, and it may be reasonable to assume that as time goes on, they will outnumber the prisoners’.^120^ This was why, throughout the 1930s, the European Resident in Lokoja proposed transferring mentally ill persons to prisons in Zaria and Kano. However, the government rejected these proposals.^121^ This continued into the 1940s. The Europeans within the asylum vicinity continued the struggle to remove the asylum. It was to be funded from the colonial development fund, only for the acting secretary to respond to the memo of the director of medical and health services that ‘it would appear that the scope of the scheme for a Lunacy Asylum for the Northern provinces has not been fully understood’.^122^ The idea did not bear fruit. In 1941, the Lokoja prison contained 173 people, including 101 mentally ill people,^123^ many of them used for labour.

Following the rejection of the proposed asylum by the acting secretary, the European community in Lokoja intensified their efforts to remove the mental asylum from their vicinity. A more radical proposal emerged: relocating the provincial capital from Lokoja to Okene. The government rejected the Okene site for several reasons. The area’s unfavourable climate and hilly terrain were deemed insurmountable challenges. In addition, concerns were raised about the potential for a scattered and inefficient layout, leading to a population influx that would strain resources. The deteriorating soil quality posed environmental risks, and the site’s limited commercial value further reduced its viability for development as compared to Lokoja.^124^ Undeterred, the Europeans suggested a more modest solution: moving the asylum away from the European residential area. This proposal too was met with resistance from the government.^125^ Instead of relocating the asylum, the authorities proposed constructing new residential quarters for the Europeans to distance them from the noise and disturbances emanating from the asylum. According to H. S. Bridel the secretary of Northern Nigeria in a letter to the chief secretary to the colonial government of the effort to assuage stemming tension, ‘While the Lokoja Asylum remains in its present site, however, it will be possible to erect half dozen quarters sufficiently far removed from the periodic din created by the asylums inmate’s to permit them to be occupied.’^126^ The above was even while the prison had no staff and the prisoner were made to attend to the mentally ill persons. Bridel wrote on 21 January 1947, ‘120 lunatics in the Asylum and about half that number of prisoners, mainly employed as attendant on the lunatic’.^127^

Subjected to labour without adequate care, these vulnerable individuals faced further exploitation and neglect. Bridel’s account offers a harrowing glimpse into the state of mental health care and the blurred lines between the criminal justice and healthcare systems in the mid-20th century. The asylum’s overcrowding, coupled with the reliance on untrained prison labour to care for the mentally ill, reveals a system in abject crisis. This arrangement not only compromised the well-being of the patients but also exploited the prisoners, underscoring a fundamental disregard for human dignity. Such practices indicate a deep-seated societal failure to recognise mental illness as a medical condition rather than a criminal offense, reflecting a prevailing punitive approach to a complex issue.

However, to justify the inaction on the asylum issue, the government pointed to the inclusion of a northern region asylum in the ten-year development plan. Furthermore, they argued that once the Abeokuta Asylum was completed, the patients from Lokoja could be transferred there pending when the asylum in the development plan materialises, while European residential area in Lokoja was moved from the prison cum asylum area. According to Bridel:

The chief secretary to the government conveyed his Excellency’s approval of the site of the new government residential area at Lokoja in his letter no. 3496/51 of the 15th of February, 1947, and follow a conversation between his honour and the deputy director of medical services, Dr. Cheverton, the director of medical services confirmed his undertaking to accommodate the mental patients at Lokoja in the new mental hospital at Abeokuta when that is opened in about two years’ time.^128^

The treatment of the mentally ill exemplifies a broader system prioritising economic gain over basic human compassion. The pre-colonial societies offered some form of support for those suffering mental illness, but the colonial era witnessed a sharp decline in their well-being.^129^ The development of Lokoja, the provincial capital, exposed the region to outsiders who filled skilled positions within the newly established trade centre.^130^ Indigenous people, on the other hand, were relegated to backbreaking labour requiring only physical exertion.^131^ These jobs were poorly paid, with wages vastly unequal to the value of the goods being handled (primarily high-value tropical produce). The coercive nature of this system, referred to as a ‘colonial oligopoly’, ensured compliance and perpetuated a cycle of economic gain at the expense of human dignity.^132^

## British bio-power in Northern Nigeria

The British colonisation of Northern Nigeria casts a long shadow on the region’s history. The arbitrary treatment of mentally ill persons and the underlying economic motives show neglect morphed into exploitation, leaving a lasting legacy of inadequate care for those with mental illness.^133^ This was because British colonial project in Northern Nigeria was primarily driven by economic gain and strategic control.^134^ Infrastructure development and resource extraction took precedence over the well-being of the local population. This translated into a policy of neglect in various sectors, including health care, education, and social services.^135^ The focus was on maintaining a compliant populace rather than fostering development.

Colonial prisons where mentally ill persons were kept in Northern Nigeria served as a stark example of this neglect. Designed for punishment and exploitation rather than rehabilitation,^136^ living conditions were harsh in these institutions. Hard labour was a cornerstone of the punishment system, with prisoners and mentally ill person’s forced to perform physically demanding tasks.^137^ While some semblance of a justice system existed through trials in native courts for prisoners, the case was different for the mentally ill persons and even for the prisoners, the overall goal was to deter and control dissent.^138^ The exploitation of the mentally ill took various forms, including manual labour, domestic chores, or any menial task that could benefit the prison authorities.^139^ Their ‘handiness’ became a tool for the colonial government to extract free labor from a vulnerable population.

The British administration sought to maximise resource extraction while minimising expenditure.^140^ Investing in proper health care, especially for the mentally ill, was not deemed a priority. Forced labour from ‘handy’ mentally ill persons provided a cheap and readily available solution for various tasks within the prison system, further incentivising neglect.^141^ Historical records indicate that the absence of formal psychiatry was not universal across Nigeria. The southern city of Calabar established the first asylum by 1904.^142^ This was followed by the Yaba asylum, also in the south, which opened its doors in 1907 with 48 patients.^143^ Previously, some individuals with mental illness in Southern Nigeria were sent to Sierra Leone for treatment.^144^ However, Northern Nigeria, a separate entity until the amalgamation of the southern and northern protectorates in 1914, followed a different path.^145^ Here, the year 1954 marked the beginning of psychiatric care when a psychiatric clinic was established at the General Hospital in Kaduna. This clinic specifically addressed the needs of mentally ill patients in the northern region.^146^

It is pertinent to note that this attitude was not limited to mentally ill patients. It was a general policy of the British during the colonial era. As pointed out earlier, while the Southern Nigeria Protectorate acknowledged the existence of yellow fever, a deadly mosquito-borne disease, the British in the north vehemently denied its presence. The consequences were severe, as the disease continued to spread unchecked in the North.^147^ The outright denial of a public health crisis profoundly underscores the systemic policy of neglect that permeated British colonial rule in Nigeria. The colonial government finally offered official acknowledgment of the disease only after the 1914 amalgamation of the Northern and Southern Protectorates.^148^

Furthermore, the British approach to education in Northern Nigeria which was part of the general colonial strategy differed significantly from the South. While they established a network of schools in the South, education in the North was largely left to Islamic institutions.^149^ British colonial rule in Northern Nigeria restricted educational opportunities for the populace. This was not a random act, but a calculated strategy. The authority, having witnessed educated elites in other colonies like Egypt and India challenge their rule, feared a similar uprising in Nigeria which was mostly Muslim like the aforementioned countries. An educated Northern population could analyse colonial practices, advocate for reform, and ultimately threaten British dominance. In addition, established power structures often relied on traditional leaders. Educating Northerners could have undermined their authority and disrupted the status quo. Divide-and-rule tactics were also at play, as limiting Northern education could weaken any potential for a unified nationalist movement across Nigeria.^150^ The strategy worked. The journey to Nigeria’s independence was a complex process marked by significant regional disparities and political manoeuvring. While the Southern part of the country was eager to attain self-governance, the Northern region was more hesitant. The Southern regions of Nigeria, especially the Western and Eastern, exhibited a strong desire for independence in the 1950s.^151^ This was fuelled by economic growth, educational advancement, and a growing nationalist sentiment. Leaders like Nnamdi Azikiwe and Obafemi Awolowo galvanised public opinion in support of self-determination.^152^

The Northern region of Nigeria adopted a more cautious stance towards independence compared to the South. This stemmed from several key factors. Firstly, the North lagged behind economically, raising concerns about its ability to thrive in a post-colonial environment (economic disparity). Secondly, infrastructural development and educational attainment were lower in the North, leading to anxieties about its capacity for self-governance. Finally, the predominantly Muslim North harboured distinct cultural and religious values, creating a sense of separate identity from the Christian-dominated South (cultural and religious differences). These factors collectively led the Northern leadership to prioritise regional development and stability before embracing independence.^153^ This led to regional tensions and compromises. Ultimately, the federal system proved to be a viable solution to bridge these divides and pave the way for independence in 1960.^154^

This neglect was also fuelled by racial ideologies. Bruce Hall reveals the long-standing presence of racial ideologies in West Africa, even before European colonialism. These ideologies often positioned ‘whiteness’ as superior and associated ‘blackness’ with inferiority and servitude.^155^ This pre-existing framework for racialisation provided a foundation for the injustices inflicted by European colonisers. European colonialism exacerbated existing racial divisions and introduced new forms of racialisation into West Africa.^156^ For example, Hall claims that the British often held a false impression of West African Muslims, primarily viewing their religious practices and beliefs as signs of inferiority and distinctiveness.^157^ West African Muslims were disadvantaged and denied rights as a result of discriminatory policies and practices brought about by this skewed viewpoint. The unfavourable preconceptions aided in the lack of comprehension and compassion for the varied experiences of Muslims in West Africa.^158^

Colonial authorities, driven by a sense of entitlement and racial superiority, viewed Africans solely as resources to be exploited.^159^ This entitlement stemmed from a belief in their inherent right to the land and resources of the indigenous population, justifying dispossession and forced labour.^160^ Steinmetz argues that these contrasting approaches were not arbitrary decisions, but rather the product of the colonists’ deeply ingrained psychological perceptions. These perceptions, shaped by cultural and historical factors, had profound consequences for the colonised populations.^161^ The sense of entitlement resulted in brutal exploitation and the near-destruction of the Herero and Nama communities.^162^ The insidious nature of European colonialism extended far beyond political and economic control. It permeated the very fabric of African societies, subtly altering perceptions and beliefs, particularly concerning mental health. At its heart was a deeply ingrained ideology, shared by all European colonisers that positioned Africans as inherently different from the whites, both physically and mentally. This perceived difference served as a potent justification for the exploitation of land, resources, and people. This disregard for the mental well-being of Africans extended to their exploitation as cheap labour in various industries. The legacy of British colonial neglect in Northern Nigeria continues to resonate today. The country’s mental healthcare infrastructure remains underdeveloped, and stigma surrounding mental illness persists.

## Conclusion

This study has explored the pervasiveness of a policy of neglect within the colonial administration and its devastating impact on one of the most vulnerable populations: those experiencing mental illness. It has shown how this contrasted sharply with pre-colonial traditional practices in Northern Nigeria, which, despite their limitations by modern standards, often sought to integrate individuals experiencing mental illness back into their communities through culturally nuanced approaches rooted in spiritual understanding and traditional healing.

The arrival of the British, however, fundamentally altered this landscape. Colonial policy aggressively prioritised economic gain and control, manifesting in a systematic disregard for health care, particularly mental health services. The chilling reality of convict prisons, with their harsh conditions and forced labour, became intertwined with the fate of the mentally ill. Categorised as ‘lunatics’, these individuals were stripped of due process, denied trials, and subjected to the same brutal conditions as convicts. A disturbing paradox emerged: while colonial authorities recognised them as ‘handy’ and exploited their perceived docility for manual labour to fuel colonial coffers, their basic well-being received no genuine consideration. The Northern Nigeria Central Lunatic Asylum in Lokoja stands as a stark and enduring example of this profound exploitation and neglect.

This research’s primary contribution lies in foregrounding how the instrumentalisation of mental illness directly served the colonial project’s insatiable hunger for labour and economic efficiency. By revealing this often-overlooked dimension of colonial exploitation, we deepen our understanding of the complex ways in which imperial power manipulated social and medical categories to its advantage, at immense human cost. The legacy of this colonial neglect continues to resonate today, not only in the underdeveloped mental healthcare infrastructure and persistent stigma in Northern Nigeria but also when viewed through broader comparative lenses of colonial health policies. Similar patterns of systematic neglect, driven by economic imperatives and a disregard for indigenous well-being, can be observed in the colonial management of other diseases. For example, the historical treatment of trypanosomiasis,^163^ the approaches to syphilis and its ‘whitening’ in French colonial medicine,^164^ and even the long-term implications for diseases like HIV/AIDS,^165^ all reflect how colonial priorities often sidelined comprehensive public health in favour of exploitative or control-oriented measures.^166^ This shared history of neglect underscores how deeply the colonial period’s influence continues to shape contemporary health challenges across the continent.

Moving forward, addressing these deeply embedded historical patterns is crucial. Investing significantly in modern mental healthcare services, promoting widespread public education to combat stigma, and critically acknowledging the historical roots of this problem are all vital steps. By learning from the past and recognising these uncomfortable truths, Nigeria can strive to build a future where mental illness is met with understanding, compassion, and proper care, ensuring that those experiencing mental illness are no longer exploited but supported on their path to recovery, requiring a concerted effort from government, healthcare providers, and communities alike.

## Supporting information

Itodo supplementary material Itodo supplementary material

## Footnotes

1

Z.O. Apata , British Administrative Changes and Reorganisations in Northern Nigeria 1897–1939 (Ibadan: Crest Hill Publishers, 2011), 1–50 .

2

R. Ajayi , ‘Kogi State: A Study in Political History’, in Z.O. Apata and Y. Akinwumi (eds.), The Groundwork of Niger-Benue Confluence History (Ibadan: Crest Hill Publishers Ltd, 2011), 326–334 .

3 NAK/Loko Prof/474a/1933/Annual Report of Kabba Province (1933), 1–25.

4 NAK/SNP 17/2962/1940/Annual Report on Kabba Province (1940), 9.

5 NAK/SNP 17/33151/Annual Report on Kabba Province, 1940, 28.

6 NAK/Loko Prof/474A/Annual Report in Kabba Province, 1949, 9.

7 International Labour Organization, *Forced Labour* (2023), 1–10. https://www.ilo.org/topics/forced-labour-modern-slavery-and-trafficking-persons/what-forced-labour.

8

M. Adas , Machines as the Measure of Men: Technology, Identity, and Gender in Industrializing Europe, 1770–1914 (New York: Cornell University Press, 2012), 45 .

9

R. Tiquet , ‘Challenging Colonial Forced Labor? Resistance, Resilience, and Power in Senegal (1920s–1940s).’ International Labor and Working-Class History, 93 (2018), 135–50. https://www.jstor.org/stable/26868601 .

10

P. Gilroy , Against Race: Imaging Political Culture Beyond the Color Line (Boston: Harvard University Press, 2000), 1–416 .

11

B.V. Leland , Mental and Social Disorder in Sub-Saharan Africa: The Case of Sierra Leone, 1787–1990 (New York: Greenwood Press 1991), xi–206 .

12

J.H. Sadowsky , Imperial Bedlam: Institutions of Madness in Colonial Southwest Nigeria (Oakland: University of California Press, 1999), xi, 169 14674439.

13

K. Kilroy-Marac , An Impossible Inheritance: Postcolonial Psychiatry and the Work of Memory in a West African Clinic (Berkeley: University of California Press, 2019), 288 .

14

N.O. Quarshie , ‘Spiritual Pawning: “Mad Slaves” and Mental Healing in Atlantic-Era West Africa’, Comparative Studies in Society and History, 65, 3 (2023), 475–499. doi:10.1017/S0010417523000051 .

15

T. Ochiai , ‘Madness in Colonial Sierra Leone’, Ryukoku Law Review, 50, 4 (2018), 531–553 .

16

M.M. Heaton , ‘Contingencies of Colonial Psychiatry: Migration, Mental Illness, and the Repatriation of Nigerian “Lunatics”’, Social History of Medicine, 27, 1 (2014), 41–63, 2023.

17

P. Barham , Outrageous Reason: Madness and Race in Britain and Empire, 1780–2020 (Monmouth: PCCS Books, 2023), p. xv11 + 248. 10.1111/bjp.12907 .

18

L.-A. Monk , ‘Exploiting Patient Labour at Kew Cottages, Australia, 1887–1950’, British Journal of Learning Disabilities, 38, 2 (2010), 86–94 .

19 S. A. Pinto, ‘Shackled Bodies, Unchained Minds: Lunatic Asylums in the Bombay Presidency 1793–1921’ (unpublished PhD dissertation, Victoria University of Wellington, 2017), https://doi.org/10.26686/wgtn.17060603.v1.

20

J. Wallis , ‘“Atrophied”, “Engorged”, “Debauched”: Muscle Wastage, Degenerate Mass and Moral Worth in the General Paralytic Patient’. Insanity and the Lunatic Asylum in the Nineteenth Century (London and New York: Routledge, 2015), 115–130 .

21

S. Snelders , ‘Leprosy and Forced Labour: Fears and Responses of the Colonial Regime in Suriname’, in Social Aspects of Health, Medicine and Disease in the Colonial and Post-colonial Era, edited by Henk Menke , Jane Buckingham , Farzana Gounder , Ashutosh Kumar , and Maurits S. Hassankhan (London and New York: Routledge, 2020), 185, 10.4324/9781003140597.

22

P. Beresford and D. Rose , ‘Decolonising Global Mental Health: The Role of Mad Studies’, Cambridge Prisms: Global Mental Health, 10 (2023), e30 37854430 10.1017/gmh.2023.21PMC10579658. Heaton, op. cit. (note 16), 41–63.

23 Steven. Metiboba, Interview BY Unekwu Friday Itodo. 70+. Professor of Medical Sociology. Anyigba. 14/10/2022.

24 W. Enoh, Friday. Interview. Conducted by Unekwu Friday Itodo 19 May 2022. Ethno-Medical Practitioner (specializing in mental health and descendant of Pa Wada Enoh).

25 O. Oboni. Interview by Unekwu Friday Itodo June 27, 2025. Ogugu District, Igala Division. Former Colonial Sanitary Officer. 90+.

26

J. Boston , ‘Medicines and Fetishes in Igala’, Africa: Journal of the International African Institute, 41, 3 (1971), 200–207. https://www.jstor.org/stable/1158838 .

27 W. Enoh, Friday. Interview. Conducted by Unekwu Friday Itodo 19 May 2022. Ethno-Medical.

28 Oboni, *op. cit.* (note 25).

29

Z.O. Apata , British Administrative Changes and Reorganisations in Northern Nigeria 1897–1939 (Ibadan: Crest Hill Publishers, 2011), 1–53 .

30
*Ibid.*, 1–53.

31 NAK/SNP 7/404/Report Annual Bassa Province, 1910, 3.

32 NAK/SNP 7/3711/Bassa Province Annual Report for June quarterly, 1909, 8.

33

J.-L. Sanchez , ‘The Penal Colonization of French Guyana 1852–1953’, HAL Open Science (2016), 1–5. shs.hal.science/halshs-01409186v1/document .

34

W. Rodney , How Europe Underdeveloped Africa (Washington, D.C.: Howard University Press, 1972), 1–30 .

35

F. Cooper , Decolonization and African Society: The Labor Question in French and British Africa (Cambridge: Cambridge University Press, 1996), 1–450 .

36 A. Gwaindepi, ‘Taxation in Africa since Colonial Times’, *African Economic History Network Working Paper* (2023), 1–15.

37

M.S. Abdulkadir , ‘Economic History of Igalaland During the Depression: 1929–1939’, in Z.O. Apata and Y. Akinwumi (eds.), The Groundwork of Niger-Benue Confluence History (Ibadan: Crest Hill Publishers Ltd, 2011), 147 .

38 NAK/Loko Prof/Q08B/1927, 1–6.

39 Abdulkadir, *op. cit.* (note 37), 15.

40

V.Y. Mudimbe , The Invention of Africa: Gnosis, Philosophy, and the Order of Knowledge (Bloomington: Indiana University Press, 1988), 1–255 .

41

E.W. Said , Orientalism (New York: Pantheon Books, 1978), 1–368 .

42

D.A. Sklansky , ‘The Private Life of Public Order: The Prison and the Problem of Punishment in Colonial Kenya’, Law and History Review, 26, 1 (2008), 1–45 .

43 NAK/ SNP 17/Conversion of Lokoja Goal (1927), 2.

44 NAK/SNP 7/1218/1907/ Report, Annual Medical, for the Year (1906), 7.

45 M. Bashir Salau, “Convict Labour in Early Colonial Northern Nigeria: A Preliminary Study,” In From Dust to Digital, édité par Maja Kominko. Cambridge: Open Book Publishers, 2015. https://books.openedition.org/obp/2246.

46

O.A. Faleye , ‘Housing “Lunatics” in Nigeria: A Study in the History of Eco-psychiatry and Psychiatric Epidemiology’, Romanian Journal of History and International Studies *(RJHIS)* , 4, 2 (2017), 137 .

47

O. Gureje , ‘Psychiatry in Nigeria’, International Psychiatry, 1, 2 (2003), 10–12 PMC673523731507663.

48
*Ibid.*,10–12.

49

F. Toyin , The History of Nigeria Greenwood Histories of Modern Nations. (London: Bloomsbury, 2002), 1–296 .

50

L.A. Abdullahi , ‘African Construction of Colonial Medicine: The Sokoto People’s Perception and Response to the British Healthcare Programme’, Sociological International Journal, 3, 5 (2019), 362–367. doi: 10.15406/sij.2019.03.00200 .

51 Welcome Collection. Annual Medical Report/Northern Nigeria. Contributors Northern Nigeria. Medical Department. Publication/Creation London: Waterlow (1913), 39. https://wellcomecollection.org/works/ehzt7wcq 39.

52 Salau, *op. cit.* (note 45), 293–329. books.openedition.org/obp/2246#ftn103.

53 NAK/ SNP 7/3711/1909/ Bassa Province Report for the Quarter 1909 (1909), 7.

54 NAK/ SNP 9/1917/Lunacy Ordinance, Section 13 and 17. (1917), 1–15.

55
*Ibid.*, 1–15.

56
*Ibid.*, 1–15.

57 NAK/ SNP10/177P/1918. Lunatic, Alledged Homicide, Onuche Aini, Transfer of, from Ankpa to Lokoja (1919), 23.

58
*Ibid.*, 23.

59 NAK/Loko Prof /179/1925/Inspection Notes by Medical and Sanitary Officers Instructions (1925), 16.

60 NAK /Loko Prof/1922/11/Koton Karfe Division Annual Report (1922), 16.

61 NAK/Loko Prof/3B/1923/Kabba Province Annual Report No: 2 for the Year Ending December (1922), 44.

62
*Ibid.*, 45.

63 NAK/Loko Prof 284/1926/Kabba Province Annual Report (1926), 8.

64 NAK/Loko *op. cit.* (note 61), 46.

65

D.O. Ali , ‘The Role and Impact of Transportation to the Socio-Economic Development of the Colonial Government of Nigeria (1900–1960)’, Journal of Sustainable Development 14, no. 5 (2021), 1–17 .

66 NAK/Lugard, Frederick John Dealtry. *Northern Nigeria Report for the Period from 1st January, 1900, to 31st March, 1901.* Colonial Reports – Annual. No. 346. London: Government Printing Office (1902), 1–12.

67

M.K. Amihera , ‘Lokoja as an Important Pre-Colonial and Colonial Trade Center’, in Z.O. Apata and Y. Akinwumi (eds.), The Groundwork of Niger-Benue Confluence History (Ibadan: Crest hill publishers, 2011), 183–3.

68 NAK/Loko, *op. cit.* (note 61), 46.

69 NAK/Loko Prof/179/1925/Inspection Note by Medical and Sanitary Officers of Instructions and Recommendation (1925), 1.

70 NAK/Loko Prof/284/1926/Kabba Province Annual Report (1926), 22.

71

F.G. Alexander and S.T. Selesnick , The History of Psychiatry: From the Stone Age to the 19th century (New York: Harper & Row, 1966), 1–471 .

72 Annual Medical and Health Report (Lagos: Government Printer, 1950), Wellcome Collection, wellcomecollection.org/works/sns2p874.

73

H. Tilley , Africa as a Living Laboratory: Empire, Development, and the Problem of Scientific Knowledge, 1870–1950 (Chicago: University of Chicago Press, 2011), 1–520 .

74

H. Tilley , ‘Medicine, Empires, and Ethics in Colonial Africa’, AMA Journal of Ethics, 18, 7 (2016), 719–729 10.1001/journalofethics.2016.18.7.mhst1-160727437825.

75

M. Vaughan , Curing Their Ills: Colonial Power and African Illness (Stanford, CA: Stanford University Press, 1991), 1–224 .

76

U.F. Itodo , ‘A History of Exploitation of the Colonised in Northern Nigeria During the Influenza Pandemic of 1918–1919’, African Historical Review, 54, no. 2 (2023), 13–36. 10.1080/17532523.2024.2314401 .

77 NAK.SNP 17/12224/Extract from Annual Report on the Prisons Department, northern Nigeria for the year 1928. (1928), 16.

78 NAK/SNP 17/12235/1941/Inspection Notes on Lokoja Prison by Senior Health Officer, 3–7 January, 1941 (1941), 2.

79 NAK/ SNP 17. 287/Lunatic Asylum (1922), 310.

80 NAK/SNP 17/Letter from Secretary Northern Provinces to Director of Prisons Northern Province, 3 September (1923), 1.

81 NAK/SNP 17/12235/Vol. 1/Lokoja Goal and Lunatic Asylum (1927), 8.

82 NAK/SNP 17. 12235/Vol. 1/Conversion of Lokoja Goal. SNP 17 (1927), 2.

83 NAK/SNP 17/12235. Vol/Lokoja Goal and Lunatic Asylum (1927), 3.

84
*Ibid.*, 3.

85 NAK/SNP 17/12235. Vol. 1/Lokoja Goal and Lunatic Asylum (1927), 3.

86
*Ibid.*, 4.

87 NAK/SNP 17/12235/Vol. 1/Lokoja Goal and Lunatic Asylum (1927), 8.

88 NAK/SNP 10/1919/ ‘Letter from Captain Bying Hall to the Resident’, *Lunatic, Alledged Homicide, Onuche of Aihi-Transfre from Ankpa to Lokoja* (1919), 11.

89 NAK/SNP. 17/12235/Lokoja Lunatic Asylum 1, 7.

90 NAK/Loko Prof/3B/1923/Kabba Province Annual Report. No 2 for the Year Ending December (1922), 44.

91
*Ibid.*, 44.

92 Arewa House Archive (AHA)/Annual Report of the Northern Provinces for the Year (1929), 20.

93 NAK/Loko Prof/10.1928/Annual Report, Idah Division (1927), 104.

94 Arewa House Archive (AHA)/19/1/1.5 AR.2/5/Annual Report of the Northern Provinces (1934), 27.

95 AHA/19/1/a.7/AR.2/7/Annual Report on the Northern Provinces (1936), 28.

96 AHA/19/1/A. R/AR2/Annual Report on the Northern Provinces (1935), 30.

97 NAK/SNP 7/12224/Lunatic Asylum for The Northern Province (1936), 18.

98 NAK/SNP 17/12268/1936/Lunatics in Northern Nigeria Provinces (1939), 490.

99 NAK/SNP 10/177P/1918/Lunatic Alleged Homicide (1918), 13.

100 A. Ichaba. Interview. 68. Professor of History, Kogis state University, Anyigba. 10 June2014.

101 Vaughan, *op. cit.* (note 75), 150–165.

102

S. Paul , ‘Prison Personnel in the Colony of Natal from circa 1850 to the Prison Reform Commission of 1905-1906’, Potchefstroom Electronic Law Journal, 26, 1 (2023), 1. doi:10.17159/1727-3781/2023/v26i0a15896 .

103

C. Van Onselen , African Mine Labour in Southern Rhodesia, 1900–1933 (Johanesbourg: Pluto Press, 1976), 1–326 .

104

O. Okia , Communal Labor in Colonial Kenya: The Legitimization of Coercion, 1912–1930 (New York: Palgrave Macmillan, 2012), 9–22 .

105

W. Rodney , How Europe Underdeveloped Africa (Verso, new edition, 2018), 259 .

106 Okia, *op. cit.* (note 104), 9–22.

107 NAK/SNP 17/12235: 1/1937/ Letter from the Senior Medical Officer, Lokoja to the Assistant Director Medical Services, Kaduna (1937), 1.

108 NAK/SNP 17/12235/vol. 2/1936/Unification and Staffing of the Colonial Prison Services (1936), 63.

109 NAK/SNP 17/12235/vol. 2/1938/Inspection Notes by Director of Medical Services (1938), 1.

110
*Ibid.*, 2.

111 NAK SNP 17/12235.2/Non-Criminal Lunatics (1936), 1–33.

112 NAK/SNP 17/12235: 2/1936/Criminal Lunatics (1936), 53.

113 NAK. Loko Prof/ 1927/206/1930/Kabba Province Annual Report (1930), 10

114 NAK/SNP 17/ 12235, 1938/ Conversion of Lokoja Goal. (1938), 1–9.

115 NAK/SNP 17/ 12235/1941/Inspection Notes on Lokoja Prison by Senior Health Officer, 3–7 January 1941 (1941), 2.

116 NAK/ SNP. 17/12235 v.2. Inspection Note on Lokoja Asylum by J. Hampton (1941), 73.

117
*Ibid.*, 73.

118 NAK/SNP 17/12235: 2/1941/Inspection Notes on Lokoja Prison by Senior Health Officer (1941), 1–2.

119 NAK/SNP 17/ 12235/1942/Extract from Notes on a Tour in Benue, Kabba and Adamawa Provinces August–September 1941 by Chief Commissioner Northern Provinces (1942), 2, 1–12.

120 NAK/SNP 17/12235: 2, 1936/Non-Criminal Lunatics. (1936), 1–33.

121
*Ibid.*, 1–33.

122
*Ibid.*, 1–14.

123
*Ibid.*, 1–14.

124 SNP 17/12235 V.2/Headquarter of Kabba Province (1947), 2.

125
*Ibid.*, 3.

126 SNP 17/12235 V.2/Prison Department Development Plan (1947), 2.

127
*Ibid.*, 1.

128
*Ibid.*, 3.

129 Abdulkadir, *op. cit.* (note 37), 146.

130

A.R. Mohammed , ‘Colonialism, State and The Spread of Islam in the Niger-Benue Confluence Area c. 1900-1960’, in Z.O. Apata and Y. Akinwumi (eds.), The Groundwork of Niger-Benue Confluence History (Ibadan: Crest Hill Publishers Ltd, 2011), 364 .

131 Amihera, *op.cit.* (note 67), 183–3.

132
*Ibid.*, 183–3.

133 NAK/SNP 17/12235/Vol. 1/Lokoja Goal and Lunatic Asylum (1927), 9.

134

T. Falola , ‘Britain and Nigeria: Exploitation or Development?’, The International Journal of African Historical Studies, 22, 1 (1989), 147–49. www.jstor.org/stable/219248.

135

K.E. Orji , ‘The Political Economy of Infrastructural Development in Nigeria’. LWATI: A Journal of Contemporary Research, 9, 1 (2012), 217–26.

136

A.I. Agbaje , ‘Prison System in Colonial Nigeria: A Historical Analysis’, International Journal of Humanities and Social Science Studies, 5, 1 (2017), 12–25 .

137
*Ibid.*, 12–25.

138
*Ibid.*, 12–25.

139 NAK/SNP 17/12235.2/non-criminal lunatics (1936), 1–33.

140

F. Toyin , Understanding Colonial Nigeria (Cambridge: Cambridge University Press, 2025), 254–275. Doi:10.1017/9781009337205 .

141

D.N. Uchegbu and A.A. Odejide , ‘Mental Health Stigma: A Review of the Literature on Prevalence, Causes, and Interventions in Nigeria’, African Journal of Psychiatry and Mental Health, 16, 4 (2017), 238–249 .

142 Gureje, *op. cit.* (note 47), 10–12.

143 C. O. Oshodi, *Psychiatry: Beginning and future in the Northern States of Nigeria* [Paper presentation. Annual Meeting of the Association of Psychiatrists in Nigeria. (1971).

144 Gureje, *op. cit.* (note 47), 10–12.

145 T. N. Tamuno. *The Evolution of the Nigerian State: The Southern Phase, 1898–1914* (Ibadan: Ibadan History Series, 1972), xvi, 422.

146 T. Asuni, “Aro Hospital in Perspective,” *American Journal of Psychiatry* 124, no. 6 (December 1967), 763. https://doi.org/10.1176/ajp.124.6.763.

147 Welcome Collection. Annual medical report/Northern Nigeria. Contributors Northern Nigeria. Medical Department. Publication/Creation London: Waterlow, (1912). Persistent URL https://wellcomecollection.org/works/p9g86fkq.

148 Wellcome Collection. Annual medical report/Northern Nigeria. Contributors Northern Nigeria. Medical Department. Publication/Creation London: Waterlow, (1914), 9. Persistent URL https://wellcomecollection.org/works/j9g85b9b.

149

S.S. Bryan , Recollections of British Administration in the Cameroons and Northern Nigeria, 1921–1957: But Always as Friends (Durham: Duke University Press, 1969), xvii–460 .

150 D. Ogun, “Sanusi Lamido Sanusi’s Question: Are We Truly Ready to Develop and Unite Nigeria?,” *Modern Ghana*, March 29, 2011, https://www.modernghana.com/news/322396/sanusi-lamido-sanusis-question-are-we-truly-ready-to-devel.html

151

N. Azikiwe , My Odyssey (London: Frank Cass & Co., 1970), 1–452 .

152

O. Awolowo , Path to Nigerian Freedom (London: Faber & Faber, 1947), 1–137 .

153 J.P. Mackintosh, ‘Nigeria since Independence’, *The World Today*, 20, 8 (August 1964), 328–37.

154
*Ibid.*

155

B.S. Hall , A History of Race in Muslim West Africa, 1600–1960. African Studies Series, Number 115 (Cambridge University Press, 2011), 30–45 .

156
*Ibid.*, 50–70.

157
*Ibid.*, 80–95.

158
*Ibid.*, 170–180.

159

G. Steinmetz , The Devil’s Handwriting: Precoloniality and the German Colonial State in Qingdao, Samoa, and Southwest Africa (Chicago: University of Chicago Press, 2007), 130–145 .

160
*Ibid.*, 150–165.

161
*Ibid.*, 170–185.

162
*Ibid.*, 190–205.

163

G. Lachenal , The Lomidine Files: The Untold Story of a Medical Disaster in Colonial Africa (Baltimore: Johns Hopkins University Press, 2017), 237 .

164 G. Linte, ‘Syphilis, Blanchiment and French Colonial Medicine in Sub-Saharan Africa during the Interwar Period’, *Medical History*, 67, 4 (2023), 307–323. doi:10.1017/mdh.2023.29.

165

M. Ndao , ‘Les prises en charge des pathologies vénériennes par le pouvoir colonial au Sénégal, 1850–1960. Eclairages et enseignements pour le sida’, Annales de la Faculté des Lettres et Science Humaines, 39/B (2009), 40–68 .

166

R. Headrick , Colonialism, Health, and Illness in French Equatorial Africa, 1885–1935 (Atlanta: African Studies Association Press, 1994), 1–495 ; and M.J. Echenberg , Black Death, White Medicine: Bubonic Plague and the Politics of Public Health in Colonial Senegal, 1914–1945 (Portsmouth: Heinemann; Oxford: James Currey, 2002), 1–328 .

## Supplementary material

The supplementary material for this article can be found at http://doi.org/10.1017/mdh.2025.10045.

