# Supplementary material for: Mental illness, forced labour, and colonial biopower in Kabba Province of Northern Nigeria, 1900–1947
Source: Med Hist. 2026 Apr;70(2):236–55. doi: 10.1017/mdh.2025.10045 (PMC13121796; doi:10.1017/mdh.2025.10045)
Supplement: Itodo supplementary material [file S0025727325100458sup001.docx]

**Appendix A: Supplementary Data**

**Table 1: Mentally Ill Persons Admitted to the Lokoja Goal and Lunatic Asylum in 1925**

|  | Name | Domicile or of what district | Criminal or non-criminal | Dangerous or non-Dangerous |
| --- | --- | --- | --- | --- |
| S852 | Utoro | Munshi | Criminal | Not |
| S104 | Onoko | Igbirra | - | - |
| 2926 | Emoru | Muye | - | - |
| S190 | Jeyin | Munshi | - | - |
| S200 | Naku | Wurkum | - | - |
| U31 | Achive | Munshi | - | - |
| U25 | Oche | Okpotor | - | - |
| V31 | Chunde | Ungwan Zaria | - | - |
| V47 | Biot Alias Mbut | Berum | - | - |
| W19 | Dan bornu | Bornu | - | - |
| W54 | Dan Geriko | Geriko | - | - |
| W98 | Dzwan | Munshi | - | - |
| W99 | Makwere (female) | - | - | Dangerous |
| W100 | Batu | - | - | Not |
| W101 | Akana | - | - | - |
| Y58 | Kaka | - | - | - |
| Y73 | Malaika | Dankerikeri | - | - |
| Y75 | Ngu | Munshi | - | - |
| Y83 | Amiye (female) | Igara | - | Dangerous |
| Y94 | Aparuwa | - | - | Not |
| Y111 | Kitjani | Munshi | - | Dangerous |
| R90 | Alhaji Sennusi | Offa | Non-criminal | Not |
| T79 | Audu Ningi | Ningi | - | - |
| V37 | Audu Maigari | Kano | - | - |
| X135 | Matan sarkin(female) | Lokoja | - | - |
| X165 | Adamu | Damagudu | - | Dangerous |
| Y80 | Ebba | Igbirra | - | Not |
| Y84 | Awjaw domo | Igara | - | - |
| Y85 | Obiagila | Kabba | - | Dangerous |
| Y87 | Arogbe | Bassa | Not |  |

Source: NAK Lokoja Goal and Lunatic Asylum. SNP 17. 12235. Vol. 1. 1927. P.9

This table lists the names, domiciles, criminal status, and dangerousness of individuals admitted to the asylum in 1925.

**Table 2: Non-Criminal Mentally Ill Persons Admitted to the Lokoja Goal and Lunatic Asylum in 1936**

| No | Name | Tribe | Date of admission | Sex |
| --- | --- | --- | --- | --- |
| T79 | Audu Ningi | Hausa | 28/09/1921 | Male |
| Y87 | Arogbe | Bassa | 22/07/1926 | - |
| Z8 | Wenizu | Igbo | 15/02/1027 | Female |
| A13 | Filani | Filani | 14/02/1928 | - |
| C14 | Moma | Beri Beri | 8/03/1930 | Male |
| C45 | Sumaila | Fulani | 15/10/- | - |
| D16 | Umoru Bussa | Hausa | 09/05/1931 | - |
| D21 | Moma | Igala | 20/06/1931 | - |
| D44 | Iddrisu of Kendi | Hausa | 17/10/1931 | - |
| E4 | Audulai | Igala | 21/01/1932 | - |
| F60 | Otitudun | Yoruba | 28/03/1933 | - |
| F67 | Abdulahi Kumasi | Wnagara | 15/05/1933 | - |
| F80 | Davies Nevins | Liberia | 23/07/1933 | - |
| F94 | Amina Oworo | Igbo | 18/10/1933 | Female |
| G10 | Adabi 0f Okene | Igbira | 15/02/1934 | Male |
| E38 | Wanjande | Munshi | 28/08/1932 | - |
| G24 | Dadi of Karu | Hausa | 17/06/1934 | - |
| G25 | Dogo of Dama | - | 17/06/1934 | - |
| G26 | Gawi of Takum | Munshi | 08/07/1934 | - |
| G29 | Fatima | Nupe | 01/08/1934 | Female |
| G35 | Mohammed Wurodole | Bamenda | 18/09/1934 | Male |
| G39 | Ilegune | Igala | 20/11/1934 | - |
| G40 | Gabriel | Igbo | 20/11/1934 | - |
| H3 | Efforo of Ezze | - | 14/01/1935 | - |
| H20 | Enegbe of Okene | Igbirra | 19/02/1935 | - |
| H15 | Gogo of Ilorin | Yoruba | 13/02/1935 | Female |
| H16 | Asta Yola | Fulani | 13/02/1935 | - |
| H17 | Jibril Kanan | - | 13/02/1935 | Male |
| H19 | Alahasan Sokoto | Hausa | 19/02/1935 | - |
| H41 | Joseph Enugu | Igbo | 18/03/1935 | - |
| H43 | Moma Sani | Hausa | 04/04/1935 | - |
| H46 | Oye Of Idape | Igbirra | 26/04/1935 | - |
| H47 | Abani of Dekina | Beri Beri | 26/04/1935 | - |
| H48 | Audu Abeokuta | Yoruba | 24/05/1935 | - |
| H104 | Anizo Nzekwe | Igbo | 28/08.1935 | - |
| H55 | Kaura Pauwa | Hausa | 25/05/1935 | - |
| H105 | M.T. Benson | Igbirra | 30.08.1935 | - |
| H125 | Adejoh of Idah | Igala | 30/10/1935 | - |
| H127 | Madaki of Gudu | - | 06/11/1935 | - |

Source: NAK. Non-Criminal Lunatics, SNP 17. 12235.2. 1936.p.1-33

This table lists the names, tribes, dates of admission, and sexes of non-criminal individuals admitted to the asylum in 1936.

**Figure 1: Map of Kabba Province**


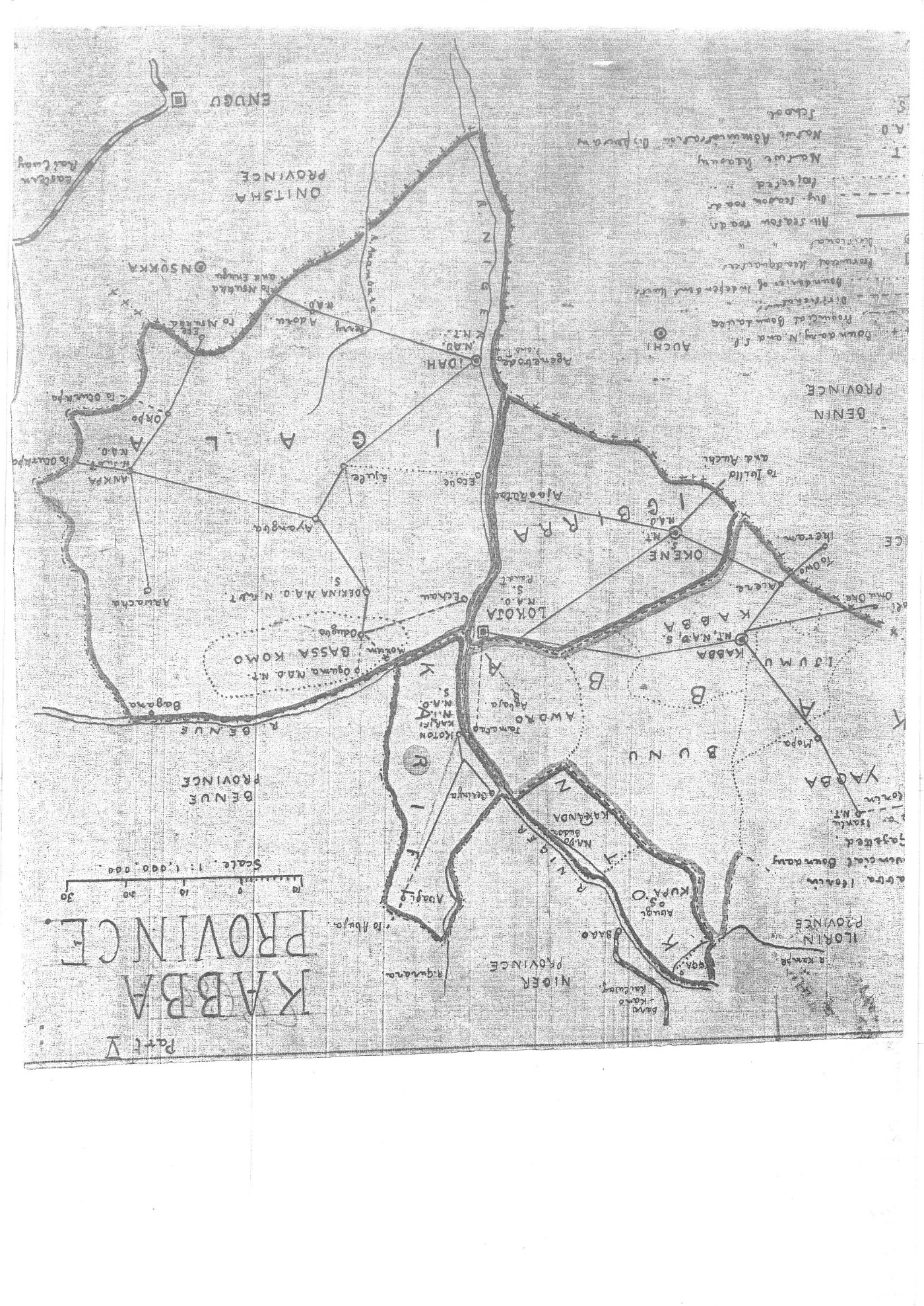


Source: NAK/ SNP 17/23,297/Kabba Province Annual Report, 1934

**Bibliography**

**Oral Interviews**

Abdulkadir, Abubakar Salisu A. Interview by Unekwu Friday Itodo. Anyigba. 2024.

Enoh, Friday Wada. Interview by Unekwu Friday Itodo. May 19, 2022.

Ichaba, Abiye. Interview by Unekwu Friday Itodo. Anyigba. June 10, 2014.

Metiboba, Steven. Interview by Unekwu Friday Itodo. Anyigba. October 14, 2022.

Oboni, Ogala. Interview by Unekwu Friday Itodo. Ogugu District, Igala Division. June 27, 2025.

Ogbebi, Shehu. Interview by Unekwu Friday Itodo. Umomi. June 30, 2025.

**Archival Documents**

**Arewa House Archive (AHA)**

AHA/19/1/1.5 AR.2/5/. Annual Report of the Northern Provinces. 1934.

AHA/19/1/A. R/AR2/. Annual Report on the Northern Provinces. 1935.

AHA/19/1/a.7/ AR.2/7/. Annual Report on the Northern Provinces. 1936.

AHA/. Annual Report of the Northern Provinces for The Year. 1929.

**National Archives Kaduna (NAK)**

NAK/SNP 7/1218/1907/. Report, Annual Medical, for the Year. 1906.

NAK/SNP 7/3711/1909/. Bassa Province Report for the Quarter. 1909.

NAK/SNP 7/12224/. Lunatic Asylum for The Northern Province. 1936.

NAK/Loko Prof/1922/11/. Koton Karfe Division Annual Report. 1922.

NAK/Loko Prof/ 3B/1923/. Kabba Province Annual Report No: 2 for the Year Ending December. 1922.

NAK/Loko Prof/ 3B/1923/. Kabba Province Annual Report No 2 for the Year Ending December. 1922.

NAK/Loko Prof/ 3B/1923/. Kabba Province Annual Report No 2 for the Year Ending December. 1922.

NAK/Loko Prof/ 3B/1923/. Kabba Province Annual Report No 2 for the Year Ending December. 1922.

NAK/Loko Prof/ 3B/1923/. Kabba Province Annual Report No 2 for the Year Ending December. 1922.

NAK/Loko Prof/3B/1923/. Kabba Province Annual Report. No 2 for the Year Ending December. 1922.

NAK/Loko Prof/3B/1923/. Kabba Province Annual Report. No 2 for the Year Ending December. 1922.

NAK/SNP 17. 287/. Lunatic Asylum. 1922.

NAK/SNP 17/. Letter from Secretary Northern Provinces to Director of Prisons Northern Province, 3rd September. 1923.

NAK/Loko Prof/179/1925/. Inspection Notes by Medical and Sanitary Officers Instructions. 1925.

NAK/Loko Prof/ 179/1925/. Inspection Note by Medical and Sanitary Officers of Instructions and Recommendation. 1925.

NAK/Loko Prof 284/1926/. Kabba Province Annual Report. 1926.

NAK/Loko Prof/ 284/1926/. Kabba Province Annual Report. 1926.

NAK/Loko Prof/Q08B/. 1927.

NAK/SNP 17/ 12235/ Vol. 1/. Lokoja Goal and Lunatic Asylum. 1927.

NAK/SNP 17/ 12235/ Vol. 1/. Lokoja Goal and Lunatic Asylum. 1927.

NAK/ SNP 17/ 12235. Vol/. Lokoja Goal and Lunatic Asylum. 1927.

NAK/SNP 17/ 12235. Vol. 1/. Lokoja Goal and Lunatic Asylum. 1927.

NAK/SNP 17/ 12235/ Vol. 1/. Lokoja Goal and Lunatic Asylum. 1927.

NAK/SNP 17/ 12235. Vol. 1/. Conversion of Lokoja Goal. 1927.

NAK/ SNP 17/ Conversion of Lokoja Goal. 1927.

NAK/SNP 17/12224/. Extract from Annual Report on the Prisons Department, northern Nigeria for the year. 1928.

NAK/Loko Prof/10.1928/. Annual Report, Idah Division. 1927.

NAK. Loko Prof/ 1927/ 206/1930/. Kabba Province Annual Report. 1930.

NAK/Loko Prof/ 474a/ 1933/. Annual Report of Kabba province. 1933.

NAK/SNP 17/ 12235: 2/. Non-Criminal Lunatics. 1936.

NAK/SNP 17/ 12235: 2/. Non-Criminal Lunatics. 1936.

NAK/SNP 17/ 12235: 2/. Non-Criminal Lunatics. 1936.

NAK/SNP 17/ 12235: 2/. Criminal Lunatics. 1936.

NAK/SNP 17/12235/vol.2/. Unification and Staffing of the Colonial Prison Services. 1936.

NAK/SNP 17/ 12235: 1/. Letter from the Senior Medical Officer, Lokoja to the Assistant Director Medical Services, Kaduna. 1937.

NAK/SNP 17/12235/vol.2/. Inspection Notes by Director of Medical Services. 1938.

NAK/SNP 17/ 12235/. Conversion of Lokoja Goal. 1938.

NAK/Loko Prof/ 1347/1939/. Juju Practice in Igala Division. 1939.

NAK/SNP 17/12268/1936/. Lunatics in Northern Nigeria Provinces. 1939.

NAK/. SNP 17/ 2962/ 1940/ Annual Report on Kabba Province. 1940.

NAK/SNP 17/33151/. Annual report on Kabba Province. 1940.

NAK/SNP 17/ 12235/ 1941/. Inspection Notes on Lokoja Prison by Senior Health Officer, 3rd -7th January. 1941.

NAK/SNP 17/ 12235/. Inspection Notes on Lokoja Prison by Senior Health Officer, 3rd -7th January. 1941.

NAK/ SNP. 17/ 12235 v.2. Inspection Note on Lokoja Asylum by J. Hampton. 1941.

NAK/SNP 17/ 12235: 2/. Inspection Notes on Lokoja Prison by Senior Health Officer. 1941.

NAK/SNP 17/ 12235/ 1942/. Extract from Notes on a Tour in Benue, Kabba and Adamawa Provinces August-September 1941 by Chief Commissioner Northern Provinces. 1942.

NAK/Loko Prof/474A/. Annual Report in Kabba Province. 1949.

NAK/SNP 10/177P/ 1918/. Lunatic Alleged Homicide. 1918.

NAK/SNP 10/177P/1918. Lunatic, Alledged Homicide, Onuche Aini, Transfer of, from Ankpa to Lokoja. 191.

NAK/ SNP 10/ 1919/. "Letter from Captain Bying Hall to the Resident." Lunatic, Alledged Homicide, Onuche of Aihi-Transfre from Ankpa to Lokoja. 1919.

NAK/SNP 9/1917/. Lunacy Ordinance, Section 13 and 17. 1917.

NAK/SNP.17/12235/. Lokoja Lunatic Asylum. n.d.

SNP 17/ 12235 V.2/. Headquarter of Kabba Province. 1947.

SNP 17/ 12235 V.2/. Prison Department Development Plan. 1947.

Wellcome Collection. Annual Medical Report / Northern Nigeria. Contributors Northern Nigeria. Medical Department. London: Waterlow, 1912. https://wellcomecollection.org/works/p9g86fkq.

Wellcome Collection. Annual Medical Report / Northern Nigeria. Contributors Northern Nigeria. Medical Department. London: Waterlow, 1913. https://wellcomecollection.org/works/ehzt7wcq.

Wellcome Collection. Annual Medical Report / Northern Nigeria. Contributors Northern Nigeria. Medical Department. London: Waterlow, 1914. https://wellcomecollection.org/works/j9g85b9b.

**Secondary Sources**

Abdullahi, Labbo A. “African Construction of Colonial Medicine: The Sokoto People's Perception and Response to the British Healthcare Programme.” Sociological International Journal 3, no. 5 (2019): 362–367. doi: 10.15406/sij.2019.03.00200.

Abdulkadir, Mohammed S. “Economic History of Igalaland During the Depression: 1929-1939.” In The Groundwork of Niger-Benue Confluence History, edited by Z.O. Apata and Y. Akinwumi, 147. Crest Hill Publishers Ltd, 2011.

Adas, Michaael. Machines as the Measure of Men: Technology, Identity, and Gender in Industrializing Europe, 1770-1914. New York: Cornell University Press, 2012.

Agbaje, A. I. “Prison System in Colonial Nigeria: A Historical Analysis." International Journal of Humanities and Social Science Studies 5, no. 1 (2017): 12-25.

Ajayi, Rotimi. “Kogi State: A Study in Political History.” In The Groundwork of Niger-Benue Confluence History, edited by Z.O. Apata and Y. Akinwumi, 326–334. Crest Hill Publishers Ltd, 2011.

Alexander, Franz G., and Saul T. Selesnick. The History of Psychiatry: From the Stone Age to the 19th Century. New York: Harper & Row, 1966.

Ali, Danladi O. “The Role and Impact of Transportation to the Socio-Economic Development of the Colonial Government of Nigeria (1900 – 1960).” Journal of Sustainable Development, 2021, 1–17.

Amihera, M.K. “Lokoja as an Important Pre-Colonial and Colonial Trade Center.” In The Groundwork of Niger-Benue Confluence History, edited by Zachaeus Oladele Apata and Yemi Akinwumi, 183-3. Ibadan: Crest Hill Publishers, 2011.

Apata, Zachaeus Oladele. British Administrative Changes and Reorganisations in Northern Nigeria 1897-1939. Crest Hill Publishers, 2011.

Apata, Zachaeus Oladele. British Administrative Changes and Reorganisations in Northern Nigeria 1897-1939. Ibadan: Crest Hill Publishers, 2011.

Asuni, Tolani. Development in Mental Health in Nigeria with Special Reference to Western Nigeria. Proceedings of the IV World Congress of Psychiatry, 1966.

Awolowo, Obafemi. Path to Nigerian Freedom. London: Faber & Faber, 1947.

Azikiwe, Nnamdi. My Odyssey. London: Frank Cass & Co., 1970.

Barham, Peter. Outrageous Reason: Madness and Race in Britain and Empire, 1780-2020.1 Monmouth: PCCS. Books, Monmouth, UK, 2023. https://doi.org/10.1111/bjp.12907.

Bell, V. Leland. Mental and Social Disorder in Sub-Saharan Africa: The Case of Sierra Leone, 1787–1990. New York: Greenwood, 1991.2

Beresford, P, and D. Rose. "Decolonising Global Mental Health: The Role of Mad Studies." Cambridge Prisms: Global Mental Health 10 (2023): e30.

Boston, John. “Medicines and Fetishes in Igala.” Africa: Journal of the International African Institute 41, no. 3 (1971): 200–207. [suspicious link removed].

Chanock, Martin. Law, Custom and Social Order: The Colonial Experience in Malawi and Zambia. Cambridge, Mass.: Cambridge University Press, 1985.3

Cooper, Frederick. Decolonization and African Society: The Labor Question in French and British Africa. Cambridge: Cambridge University Press, 1996.4

Echenberg, Myron J. Black Death, White Medicine: Bubonic Plague and the Politics of Public Health in Colonial Senegal, 1914-1945.1 Portsmouth, NH: Heinemann; Oxford: James Currey, 2002.

Faleye, Olukayode A. “Housing ‘Lunatics’ in Nigeria: A Study in the History of Eco-psychiatry and Psychiatric Epidemiology.” Romanian Journal of History and International Studies (RJHIS) 4, no. 2 (2017): 137.

Falola, Toyin. The Colonial Economy of Nigeria. Oakland: University of California Press, 2005.

Falola, Toyin. The History of Nigeria. Greenwood Publishing Group, 2002.

Falola, Toyin. "Britain and Nigeria: Exploitation or Development?" The International Journal of African Historical Studies 22, no. 1 (1989): 147-49. www.jstor.org/stable/219248.

Gilroy, Paul. Against Race: Imaging Political Culture Beyond the Color Line. Boston: Harvard University Press, 2000.

Gureje, Oye. “Psychiatry in Nigeria.” Int Psychiatry 1, no. 2 (2003): 10–12.

Gwaindepi, Abel. "Taxation in Africa since colonial times." African Economic History Network Working Paper, 2023.

Haque, A. "Psychology and Religion: Their Relationship and Integration from an Islamic Perspective." American Journal of Islam and Society 15, no. 4 (1998): 97–116. https://doi.org/10.35632/ajis.v15i4.2143.

Hall, Bruce S. A History of Race in Muslim West Africa, 1600-1960. Cambridge University Press, 2011.

Headrick, Rita. Colonialism, Health, and Illness in French Equatorial Africa, 1885–1935. Atlanta: African Studies Association Press, 1994.

Heaton, Matthew M. "Contingencies of colonial psychiatry: migration, mental illness, and the repatriation of Nigerian ‘lunatics’." Social History of Medicine 27, no. 1 (2014): 41-63.5

International Labour Organization. Forced Labour. 2023. https://www.ilo.org/topics/forced-labour-modern-slavery-and-trafficking-persons/what-forced-labour.

Itodo, Unekwu Friday. “A History of Exploitation of the Colonised in Northern Nigeria During the Influenza Pandemic of 1918–1919.” African Historical Review, 2024, 7–18. DOI: 10.1080/17532523.2024.2314401.

Kilroy-Marac, Katie. An Impossible Inheritance: Postcolonial Psychiatry and the Work of Memory in a West African Clinic. Berkeley, CA: University of California Press, 2019.

Lachenal, Guillaume. The Lomidine Files: The Untold Story of a Medical Disaster in Colonial Africa. Baltimore: Johns Hopkins University Press, 2017.

Linte, Guillaume. « Syphilis, blanchiment and French colonial medicine in sub-Saharan Africa during the interwar period ». Medical History 67, no. 4 (2023): 307–323. doi:10.1017/mdh.2023.29.

Lugard, Frederick John Dealtry. Northern Nigeria Report for the Period from 1st January, 1900, to 31st March, 1901. Colonial Reports—Annual.6 No. 346. London: Government Printing Office, 1902.

Mackintosh, John P. "Nigeria since Independence." The World Today 20, no. 8 (1964): 328-37.

Mitha K. "Conceptualising and addressing mental disorders amongst Muslim communities: Approaches from the Islamic Golden Age."7 Transcultural Psychiatry 57, no.2 6 (2020): 763-774. doi: 10.1177/1363461520962603.

Mohammed, A.R. “Colonialism, State and The Spread of Islam in the Niger-Benue Confluence Area c. 1900-1960.” In The Groundwork of Niger-Benue Confluence History, edited by Zachaeus Oladele Apata Z.O. and Yomi Akinwumi, 364. Ibadan: Crest Hill Publishers Ltd, 2011.

Monk, Lee‐Ann. "Exploiting Patient Labour at Kew Cottages, Australia, 1887–1950." British Journal of Learning Disabilities 38, no. 2 (2010): 86-94.

Mudimbe, V. Y. The Invention of Africa: Gnosis, Philosophy, and the Order of Knowledge. Bloomington: Indiana University Press, 1988.

Ndao, Mor. « Les prises en charge des pathologies vénériennes par le pouvoir colonial au Sénégal, 1850-1960. Eclairages et enseignements pour le sida ». Annales de la Faculté des Lettres et Science Humaines 39/B (2009): 40-68.

Nigeria. Medical Department, contributor. Annual Medical and Health Report. Lagos: Government Printer, 1950. Wellcome Collection, wellcomecollection.org/works/sns2p874.

Ochiai, Takehiko. “Madness in Colonial Sierra Leone.” Ryukoku Law Review 50, no. 4 (2018): 531-553.

Ogun, Dele. “Sanusi Lamido Sanusi's Question: Are We Truly Ready to Develop and Unite Nigeria?" Modern Ghana, 2011. https://thenationonlineng.net/sanusi-goes-to-ghana/.

Okia, Opolot. Communal Labor in Colonial Kenya: The Legitimization of Coercion, 1912-1930. New York: Palgrave Macmillan, 2012.8

Onselen, Charles Van. African Mine Labour in Southern Rhodesia, 1900-1933. Johanesbourg: Pluto Press, 1976.

Orji, Kingdom E. "The Political Economy of Infrastructural Development in Nigeria." LWATI: A Journal of Contemporary Research 9, no. 1 (2012): 217-26.

Oshodi, C. O. Psychiatry: Beginning and Future in the Northern States of Nigeria. Paper presentation. Annual Meeting of the Association of Psychiatrists in Nigeria. 1971.

Pinto, Sarah Ann. Shackled Bodies, Unchained Minds: Lunatic Asylums in the Bombay Presidency 1793-1921.9 PhD diss., Open Access Te Herenga Waka-Victoria University of Wellington, 2017. file:///C:/Users/USER/Downloads/thesis_access-1.pdf.

Quarshie, Nana Osei. “Spiritual Pawning: “Mad Slaves” and Mental Healing in Atlantic-Era West Africa.” Comparative Studies in Society and History 65, no. 3 (2023): 475–499. doi:10.1017/S0010417523000051.

Rodney, Walter. How Europe Underdeveloped Africa. Washington, D.C.: Howard University Press, 1972.

Rodney, Walter. How Europe Underdeveloped Africa. New edition. Verso, 2018.

Sadowsky, Jonathan Hal. Imperial Bedlam: Institutions of Madness in Colonial Southwest Nigeria.10 Oakland: University of California Press, 1999.3

Said, Edward W. Orientalism. New York: Pantheon Books, 1978.

Salau, Mohammed Bashir. "Convict Labour in Early Colonial Northern Nigeria: A Preliminary Study." In Open, edited by Maja Kominko, 293-329. Open Book Publishers, 2017. books.openedition.org/obp/2246#ftn103.

Sanchez, Jean-Lucien. "The Penal Colonization of French Guyana 1852-1953." HAL Open Archive, 2023. shs.hal.science/halshs-01409186v1/document.

Sharwood Smith, Bryan. Recollections of British Administration in the Cameroons and Northern Nigeria, 1921-1957: But Always as Friends. Durham, N.C., Duke University Press, 1969.

Sklansky, David Alan. "The Private Life of Public Order: The Prison and the Problem of Punishment in Colonial Kenya." Law and History Review 26, no. 1 (2008): 1-45.

Snelders, Stephen. "Leprosy and Forced Labour: Fears and Responses of the Colonial Regime in Suriname." In Social Aspects of Health, Medicine and Disease in the Colonial and Post-colonial Era, 179-198. Routledge, 2020.

Steinmetz, George. The Devil's Handwriting: Precoloniality and the German Colonial State in Qingdao, Samoa, and Southwest Africa. University of Chicago Press, 2007.

Swanepoel, Paul. "Prison Personnel in the Colony of Natal from circa 1850 to the Prison Reform Commission of 1905-1906." Potchefstroom Electronic Law Journal 26, no. 1 (2023): 1. doi:10.17159/1727-3781/2023/v26i0a15896.

Tamuno, Tekena. The Evolution of the Nigerian State: The Southern Phase, 1898–1914.11 1972.

Tiquet, Romain. “Challenging Colonial Forced Labor? Resistance, Resilience, and Power in Senegal (1920s–1940s).” International Labor and Working-Class History, no. 93 (2018): 135–50. [suspicious link removed].

Tilley, Helen. Africa as a Living Laboratory: Empire, Development, and the Problem of Scientific Knowledge, 1870-1950.12 University of Chicago Press, 2011.

Tilley, Helen. "Medicine, Empires, and Ethics in Colonial Africa." AMA Journal of Ethics 18, no. 7 (2016): 719-729.

Tzeferakos, Themistoklis, and Elias Douzenis. "Islam, Mental Health and Law: A General Overview." Annals of General Psychiatry 16, no. 1 (2017): 28.

Uchegbu, D. N., and A. A. Odejide. “Mental Health Stigma: A Review of the Literature on Prevalence, Causes, and Interventions in Nigeria." African Journal of Psychiatry and Mental Health 16, no. 4 (2017): 238-249.

Vaughan, Megan. Curing Their Ills: Colonial Power and African Illness. Stanford University Press, 1991.

Wallis, Jennifer. "'Atrophied', 'Engorged', 'Debauched': Muscle Wastage, Degenerate Mass and Moral Worth in the General Paralytic Patient." In Insanity and the Lunatic Asylum in the Nineteenth Century, 115-130. Routledge, 2015.

Yakubu, B.C. Omelle. “Igala.” In The Groundwork of Niger-Benue Confluence History, edited by Z.O. Apata and Y. Akinwumi, 15. Crest Hill Publishers Ltd, 2011.
